# PCP‐B class pollen coat proteins are key regulators of the hydration checkpoint in *Arabidopsis thaliana* pollen–stigma interactions

**DOI:** 10.1111/nph.14162

**Published:** 2016-09-06

**Authors:** Ludi Wang, Lisa A. Clarke, Russell J. Eason, Christopher C. Parker, Baoxiu Qi, Rod J. Scott, James Doughty

**Affiliations:** ^1^ Department of Biology and BiochemistryUniversity of BathClaverton DownBathBA2 7AYUK

**Keywords:** *Arabidopsis thaliana*, compatibility, pollen coat proteins, pollen hydration, pollen–stigma interaction, reproduction, signalling

## Abstract

- The establishment of pollen–pistil compatibility is strictly regulated by factors derived from both male and female reproductive structures. Highly diverse small cysteine‐rich proteins (CRPs) have been found to play multiple roles in plant reproduction, including the earliest stages of the pollen–stigma interaction. Secreted CRPs found in the pollen coat of members of the Brassicaceae, the pollen coat proteins (PCPs), are emerging as important signalling molecules that regulate the pollen–stigma interaction.
- Using a combination of protein characterization, expression and phylogenetic analyses we identified a novel class of *Arabidopsis thaliana* pollen‐borne CRPs, the PCP‐Bs (for pollen coat protein B‐class) that are related to embryo surrounding factor (ESF1) developmental regulators. Single and multiple *PCP‐B* mutant lines were utilized in bioassays to assess effects on pollen hydration, adhesion and pollen tube growth.
- Our results revealed that pollen hydration is severely impaired when multiple PCP‐Bs are lost from the pollen coat. The hydration defect also resulted in reduced pollen adhesion and delayed pollen tube growth in all mutants studied.
- These results demonstrate that *At* PCP‐Bs are key regulators of the hydration ‘checkpoint’ in establishment of pollen–stigma compatibility. In addition, we propose that interspecies diversity of PCP‐Bs may contribute to reproductive barriers in the Brassicaceae.

## Introduction

Pollination in angiosperms involves multiple phases of interaction between female reproductive tissues of the gynoecium and the male reproductive unit, pollen, and subsequently the pollen tube it produces on germination (Hiscock & Allen, 2008). The process is highly selective such that the majority of heterospecific pollen fails to effect syngamy and indeed intraspecific pollination can also be blocked in those species which possess self‐incompatibility (SI) systems. Such prezygotic reproductive barriers are evolutionarily advantageous as they limit wasted mating opportunities, contribute to reproductive isolation and facilitate outbreeding when SI is present (Yost & Kay, 2009; Smith *et al*., 2013). The establishment of compatibility is complex and involves a suite of biophysical and molecular recognition factors that operate throughout the pollination process; from pollen capture by the stigma, pollen hydration, germination and stigmatic penetration, to polarized tube growth through the pistil (reviewed in Chapman & Goring, 2010). Although much progress has been made in elucidating the mechanisms that regulate compatibility in a broad range of species, it is clear that great mechanistic diversity exists, with no common system in operation (Hiscock & Allen, 2008; Allen *et al*., 2011). Such diversity is considered to be important in maintaining species barriers (Swanson & Vacquier, 2002; Takeuchi & Higashiyama, 2012).

Members of the Brassicaceae family (which includes *Brassica* and *Arabidopsis* species) are characterized by having stigmas of the ‘dry’ type, which lack sticky secretions such as those present in species of the Solanaceae (Heslop‐Harrison & Shivanna, 1977). Dry stigmas are highly discriminatory, reducing the probability that heterospecific pollen grains and pathogenic spores will be captured, hydrate and germinate on their surfaces. In this system compatibility is established at the stigma surface within minutes of pollination when compatible grains gain access to stigmatic water, but incompatible pollen generally fail to fully hydrate and germinate. Thus, pollen hydration on the stigma surface is essential for successful reproduction and is a strictly regulated checkpoint centred in the stigma (Dickinson, 1995; Ma *et al*., 2012; Hiroi *et al*., 2013). Possession of a dry stigma comes with the requirement that the exine surface of conspecific pollen must carry a coating (tryphine) (Dickinson, 1995; Dickinson *et al*., 2000). Tryphine is a complex mixture of lipids, proteins, glycoconjugates and pigments (Piffanelli *et al*., 1998; Hernandez‐Pinzon *et al*., 1999) that confers adhesive properties to the grain, provides a conduit for water to pass from the stigma to effect pollen hydration and, importantly, carries factors that determine compatibility (Dickinson, 1995; Safavian & Goring, 2013). Pollen access to stigmatic water requires targeted secretion in the stigma immediately adjacent to a compatible pollen grain (Dickinson, 1995) and it is now well established that in the Brassicaceae this involves exocyst‐mediated tethering of secretory vesicles to the stigmatic plasma membrane (Samuel *et al*., 2009; Safavian & Goring, 2013; Safavian *et al*., 2015).

Despite progress in identifying molecular regulators of compatibility in stigmas, relatively little is known about the pollen‐borne signals that establish it. Components of the pollen coat are most likely to mediate compatibility due to the intimate interaction of this layer with the surface of stigmatic papilla cells and the speed of pollen acceptance (Elleman & Dickinson, 1986, 1990; Preuss *et al*., 1993). Indeed, application of isolated pollen coat to the stigma surface evokes a rapid expansion of the stigmatic outer wall layer (Elleman & Dickinson, 1990, 1996) and isolated pollen stimulates the production of structures resembling vesicles in the stigma apoplast beneath the pollen contact site (Elleman & Dickinson, 1996).

Analysis of pollen coat components and mutational studies in *Arabidopsis thaliana* have shed light on factors that influence the pollen–stigma interaction. A number of these appear to be biophysical in nature, for example *eceriferum* (*cer*) mutant pollen fails to hydrate due to the elimination of very long chain lipids from the pollen coat (Preuss *et al*., 1993; Hulskamp *et al*., 1995; Fiebig *et al*., 2000). Hydration defects have also been reported in mutants for extracellular lipase 4 (EXL4) and GRP17, an oleosin‐domain‐containing glycine‐rich protein which may work cooperatively to alter the lipid composition at the pollen–stigma interface to facilitate the passage of water to the grain (Mayfield & Preuss, 2000; Updegraff *et al*., 2009). Work in *Brassica* has led to the identification of several groups of small cysteine‐rich pollen coat proteins (Doughty *et al*., 1993, 1998, 2000; Hiscock *et al*., 1995; Schopfer *et al*., 1999; Takayama *et al*., 2000; Shiba *et al*., 2001). Importantly these polypeptides, rather than having major effects on biophysical properties of the pollen coat, act as ligands and have been demonstrated to bind a number of stigmatic proteins.

In recent years, a broad range of cysteine‐rich proteins (CRPs) have been identified in plants having functions in cell signalling, development and defence (Silverstein *et al*., 2007; Li *et al*., 2014). They are all characterized by being small (< 160 amino acids), having a conserved N‐terminal signal peptide and a C‐terminal cysteine‐rich region with the pattern of cysteines determining their classification (Marshall *et al*., 2011). A number function in plant reproduction, including pollen–stigma self‐recognition (Schopfer *et al*., 1999; Shiba *et al*., 2001), pollen tube growth and guidance (Chae *et al*., 2009; Okuda *et al*., 2009), and early embryo development (Marshall *et al*., 2011; Costa *et al*., 2014). Several families of CRPs have been identified in the pollen coat of *Brassica* and *Arabidopsis,* and some have confirmed roles in the pollen–stigma interaction. In self‐incompatible species, the *S*‐locus cysteine‐rich protein (SCR/SP11) (Schopfer *et al*., 1999; Shiba *et al*., 2001), acts as the male determinant and interacts with the *S*‐receptor kinase (SRK) (Takasaki *et al*., 2000), to trigger pollen rejection by targeted degradation of the basal compatibility factor EXO70A1 (Stone *et al*., 2003; Samuel *et al*., 2009). Other CRPs belonging to the PCP‐A class of *Brassica* pollen coat proteins such as SLR‐BP1 and PCP‐A1, bind the stigmatic proteins *S*‐locus related 1 (SLR1) and *S‐*locus glycoprotein (SLG), respectively, and thus are likely to function in the pollen–stigma interaction, even though their precise function remains to be determined (Doughty *et al*., 1998; Takayama *et al*., 2000). A further class of *Brassica* pollen coat CRPs, the PCP‐Bs, have been described and also are good candidates for regulators of the earliest phases of the pollen–stigma interaction (Doughty *et al*., 2000). Thus, there is a growing body of evidence demonstrating that the pollen coat carries factors that mediate both incompatibility and compatibility, and that cysteine‐rich pollen coat proteins are important to molecular dialogue in the pollen–stigma interaction. Although many studies have focused on the mechanisms of self‐incompatibility (SI) in a range of species, the molecular regulation of self‐compatibility (SC) in flowering plants is still poorly understood.

In this study, we report on the identification of four *A. thaliana* PCP‐B encoding genes termed *AtPCP‐Bα* (*At5g61605*), *AtPCP‐Bβ* (*At2g29790*), *AtPCP‐Bγ* (*At2g16535*) and *AtPCP‐Bδ* (*At2g16505*), which are expressed gametophytically late in pollen development. By utilizing T‐DNA insertion lines carrying single, double and triple *AtPCP‐B* gene knockouts, we examined the impact of these mutations on pollen morphology and the pollen–stigma interaction. Phenotypic analyses revealed defects in pollen hydration and delays in pollen tube growth for single and combined mutants compared with wild‐type. Triple mutant *pcp‐bα*/*β*/*γ* pollen displayed a substantially reduced hydration rate on stigmas, delayed pollen tube growth, as well as weakened anchoring to the stigma surface. Importantly, no impact on pollen morphology was revealed in this study though the mutants presented striking effects on early post‐pollination events. Such evidence suggests the *At*PCP‐Bs act as important regulatory factors during the earliest stages of the pollen‐stigma interaction by establishing a molecular dialogue between the stigma and pollen grains.

## Materials and Methods

### Plant material and growth conditions

*Brassica oleracea* var. *alboglabra* L. homozygous for incompatibility haplotypes S25 and S29 (Horticultural Research International, Wellesbourne, UK) was used for isolation of *Bo*PCP‐B1 and *Bo*PCP‐B2 pollen coat proteins, respectively. *Arabidopsis thaliana* (L.) Heynh. T‐DNA insertion lines SALK_207087, SALK_062825, SALK_072366 (Alonso *et al*., 2003) were obtained from the Nottingham Arabidopsis Stock Centre (NASC). GABI_718B04 was purchased from GABI‐KAT (Kleinboelting *et al*., 2012). T‐DNA insertion sites and their respective mutant alleles are detailed in Supporting Information Fig. S1. Single gene T‐DNA insertion lines were backcrossed to wild‐type (WT; Col‐0) at least three times before phenotyping. *pcp‐bβ*/*γ* and *pcp‐bα*/*β*/*γ* mutant lines were created using standard crossing procedures. *PCP‐B* transcript status for each mutant was confirmed by reverse transcription polymerase chain reaction (RT‐PCR) (Fig. S2). Primers for RT‐PCR are detailed in Table S1. The A9‐barnase male sterile line was provided by Rod Scott, University of Bath, UK (Paul *et al*., 1992). GUS (β‐glucuronidase) reporter lines *pAt5g61605:GUS* and *pAt2g16505:GUS* were provided by José F. Gutierrez‐Marcos (University of Warwick, UK).

*Arabidopsis thaliana* plants were propagated in Levington F2 + S compost (Soils HS Limited, Wotton‐Under‐Edge, UK) in a controlled environment room with a 16 h : 8 h, light : dark photoperiod provided by fluorescent lighting (130 μmol m^−2^ s^−1^). Temperature was maintained at 21 ± 1°C with 60% relative humidity. Brassicas were grown in a glasshouse at 21°C with a 16 h : 8 h, light : dark photoperiod.

### RT‐PCR and RNA gel blot analysis

Anthers were collected from *A. thaliana* stage 10–12 flower buds (Smyth *et al*., 1990), stigmas from open flowers of the *A. thaliana* A9‐barnase line, roots from 2‐wk‐old seedlings grown on 0.5× MS plates and leaves from fully‐grown rosettes. RNA was extracted using a PureLink^®^ RNA Mini Kit (Thermo Fisher Scientific, Loughborough, UK). cDNA synthesis was carried out using the ProtoScript^®^ II First Strand cDNA Synthesis Kit (New England Biolabs, Hitchin, UK). DNA amplification utilized DreamTaq^®^ Green PCR Master Mix (2×) (Thermo Fisher Scientific). Primers for DNA amplification are detailed in Table S1. RNA gel‐blot analysis was carried out as described previously (Doughty *et al*., 1998) using polyadenylated mRNA (450 ng) from leaves, stigmas, pollen and anthers derived from a range of bud sizes (whole flower buds for anthers of size < 2 mm). Labelling of gene‐specific *BoPCP‐B* probes (covering the coding region of the gene from aa residue 40 to the C‐terminus) was conducted using the Prime‐a‐Gene Labeling System (Promega), with modifications to the deoxynucleotidetriphosphates mix to permit double labelling with dATP and dCTP (α‐^32^P, 100 μCi, 6000 Ci mmol^−1^ each).

### Pollen hydration assays

For *in vivo* hydration assays, pollen grains derived from WT, *pcp‐bα* (SALK_207087), *pcp‐bβ* (SALK_062825), *pcp‐bγ* (SALK_072366), *pcp‐bδ* (GABI_718B04), *pcp‐bβ*/*γ* and *pcp‐bα*/*β*/*γ* lines were applied to stigmas of the A9‐barnase male sterile line. Freshly opened mature flowers were used (retained on the plant) and pollen was applied in a monolayer with an eyelash. At least eight independent stigmas were used in assays from each line. For *in vitro* hydration assays, pollen from WT and *pcp‐bα*/*β*/*γ* triple knockout lines were placed on a slide in a humid chamber (100% relative humidity). Pollen grains on stigmas were photographed under a dissecting microscope immediately after pollinations were initiated (designated as time point zero). Subsequent images were captured every minute for 30 min. For humid chamber assays pollen was photographed 1 min after pollen grains were placed, then the chamber was sealed, after which images were taken every minute for 30 min. Equatorial diameter of pollen was measured in pixels using ImageJ software (Schneider *et al*., 2012). Pollen hydration (%) was calculated using the equation: pollen hydration (%) = (pollen diameter − initial pollen diameter)/initial pollen diameter. Slopes were determined using 11 data points during the 0–10 min, 10–20 min, or 20–30 min time periods using the linear regression curve *f *= *a*_0_ *x* + *b*. All statistical analyses were carried out using Microsoft Excel 2013.

### Pollen adhesion assay

Stigmas of *A. thaliana* A9‐barnase plants were hand‐pollinated using freshly dehiscent anthers from WT and *pcp‐bα*/*β*/*γ* lines. Pollen was applied as a monolayer. After 30 min the flower was excised from the plant and placed into 0.5 ml of fixative (60% v/v ethanol, 30% v/v chloroform, 10% v/v acetic acid) in a 1.5‐ml microfuge tube. The sample was immediately shaken 10 times using short sharp strokes to dislodge pollen that was not strongly adherent to the stigma. The flower was then removed and placed into a separate microfuge tube. Both samples were retained for pollen counting. Fifty microlitres of aceto‐orcein stain (1%) was added to the tubes and incubated overnight at room temperature (RT). ‘Washed‐off’ pollen samples were centrifuged at 13 000 ***g*** (10 min), excess fixative was removed and the pellet was resuspended in 10 μl of 50% glycerol before counting. Stigmas were excised from stained flowers before mounting in 50% glycerol and were squashed on a slide to ensure all pollen was visible for counting.

### Pollen tube growth assay

Pollinations were initiated on *A. thaliana* male sterile A9‐barnase stigmas and allowed to proceed for 2 or 4 h before stigmas were excised and incubated in fixative (60% v/v ethanol, 30% v/v chloroform, 10% v/v acetic acid) overnight. After removal of fixative, stigmas were incubated in 8 M NaOH for 20 min then washed in dH_2_O three times, each for 5 min. Samples were transferred to 0.1% decolourized aniline blue (0.1% w/v aniline blue in 0.1 M K_3_PO_4_, pH 11) for 1 h before imaging (Kho & Baer, 1968).

### Microscopy

Imaging of pollen hydration on stigmas, hydration in a humid chamber and GUS histochemical staining (Methods S1) of flowers and leaves was carried out using a Nikon (Surrey, UK) SMZ1500 dissection microscope coupled to Nikon Digital Sight DS‐U1 camera. A Nikon Eclipse 90i epifluorescence microscope (×10 objective) with Nikon Digital Sight DS‐U1 camera was used for imaging pollen tubes stained with aniline blue and for anthers stained for GUS activity.

### Scanning and transmission electron microscopy

Pollinated stigmas were dry‐fixed using the method of Elleman & Dickinson (1986). Samples were washed with 0.1 M sodium cacodylate buffer pH 7.4 and prepared for scanning electron microscopy (SEM) and transmission electron microscopy (TEM) by a modified method of Villar *et al*. (1987) using 2.5% glutaraldehyde and low viscosity resin. Samples for SEM were gold‐coated and imaged using a Jeol JSM640LV Scanning Electron Microscope (Jeol, Tokyo Japan). Samples for TEM were ultra‐thin sectioned (100 nm) on an Ultracut‐E ultramicrotome (Leica, London, UK) and imaged by Jeol JEM1200EXII transmission electron microscope.

### Multiple sequence alignment and phylogenetic analysis

*PCP‐B*‐like sequences were retrieved from available complete plant genomes completed to at least scaffold level using TBlastN database searches (Phytozome, https://phytozome.jgi.doe.gov; Comparative Genomics, CoGe, https://genomevolution.org). Nucleotide sequence alignments of *PCP‐B* homologous genes were generated using Muscle (codon) (Edgar, 2004). Codons of protein coding sequences were translated into amino acid sequences before the alignment was performed. Aligned amino acid sequences were then replaced by the original codons. Graphical output of protein sequence alignment was generated by Jalview using ‘ClustalX’ colour coding. Phylogenetic trees were built using the maximum‐likelihood statistical method in Mega v.6 (Tamura *et al*., 2013). The initial tree was determined by the neighbour‐joining method (NJ). The phylogeny test was carried out using the bootstrap method (1000 replications). Phylogenetic trees were displayed using iTOL (Letunic & Bork, 2007).

### Protein structure prediction and modelling

Protein sequence alignment of PCP‐Bs and 2RU1 was carried out with T‐Coffee (Notredame *et al*., 2000). PCP‐B protein models were built using Swiss‐model (Arnold *et al*., 2006; Guex *et al*., 2009; Kiefer *et al*., 2009; Biasini *et al*., 2014) based on the modified T‐Coffee alignment result. 3D cartoon models and electrostatic potential surface models were produced by PyMOL (v.1.7.4; Schrödinger, Cambridge, UK).

### *In situ* hybridization

Flower buds were excised from inflorescences and immediately fixed in fresh 4% paraformaldehyde for 16 h at 4°C with an initial 10 min under low vacuum. Tissues were embedded in Paraplast Xtra (Sigma), sectioned (7–10 μm) and prepared for probing as described by Langdale (1994), except for the protease treatment where sections were incubated for 30 min at 37°C in 50 μg ml^−1^ proteinase K (Sigma). Both antisense and sense probes were synthesized using a SP6/T7 digoxigenin RNA labeling kit (Boehringer Mannheim, Lewes, UK), according to the manufacturer's instructions. Probes covered the protein coding sequence of the *AtPCP‐Bβ* and *AtPCP‐Bγ* cDNAs.

### Protein purification and N‐terminal sequencing

*Bo*PCP‐B1 and *Bo*PCP‐B2 proteins were purified from total pollen coat proteins by a combination of gel filtration, RP‐HPLC and cation exchange chromatography following the protocol described by Doughty *et al*. (1993, 1998). Both *Bo*PCP‐B1 and *Bo*PCP‐B2 co‐purified with the previously characterized PCP‐A1 polypeptide following C18 RP‐HPLC and were separated by cation exchange chromatography (Doughty *et al*., 1998). *Bo*PCP‐B1 and *Bo*PCP‐B2 were isolated from *S*25 and *S29* incompatibility lines of *B. oleracea* var *alboglabra*, respectively. *Bo*PCP‐B protein samples were prepared for N‐terminal sequencing as described previously (Doughty *et al*., 1998).

### 5′ and 3′ rapid amplification of cDNA end (RACE) polymerase chain reaction cloning of *BoPCP‐B1 and BoPCP‐B2*

Polyadenylated RNA was isolated from 100 mg of anthers derived from 9 to 11 mm flower buds of *B. oleracea* var *alboglabra* (homozygous for S25 and S29 incompatibility haplotypes) using a QuickPrep Micro mRNA purification kit (Pharmacia Biotech, Piscataway, NJ, USA). Cloning of *BoPCP‐B1* and *BoPCP‐B2* cDNA sequences was carried out using a 5′/3′ RACE kit (Boehringer Mannheim) following the manufacturer's instructions. One microgram of mRNA was subjected to first‐strand cDNA synthesis using an oligo(dT) kit primer. First round 3′ RACE cloning of *BoPCP‐B1* utilized a degenerate primer based on the peptide sequence AGNAAK[P/Q] which is common to both *Bo*PCP‐B proteins (5′‐GC‐GGATCC‐GCIGGIAA[C/T]GCIGCIAA[A/G]C‐3′, where I represents inosine) in conjunction with a kit anchor primer. This was followed by two rounds of PCR using degenerate nested primers (5′‐GC‐GGATCC‐AA[A/G]CA[A/G]ACICCITG[C/T]CA[C/T]G‐3′ and 5′‐GC‐GGATCC‐AA[A/G]CCIAA[C/T]CA[C/T]ACITG) based on the *Bo*PCP‐B1 specific peptide sequences KQTPCHE and KPNHTC, respectively. 5′ RACE was conducted utilizing sequence‐specific primers SP1 and SP2 (derived from 3′RACE) for cDNA synthesis and nested amplification of the 5′ region of the *BoPCP‐B1* cDNA (SP1 5′‐GCTTGCCGCACCTACGCG‐3′ and SP2 5′‐CAT GTAGCACATGTTTTGAGC‐3′). For *BoPCP‐B2*, first round 3′RACE was carried out as described for *BoPCP‐B1* followed by one further round of PCR utilizing a degenerate nested primer (5′‐GC‐GGATCC‐ATGAA[C/T]TG[C/T]GA[C/T]ACICA[A/G] G) based on the BoPCP‐B2 specific peptide sequence MNCDTQD. 5′ RACE utilized sequence‐specific primers SP1 and SP2 (derived from 3′RACE sequence) for cDNA synthesis and nested amplification of the 5′ region of the BoPCP‐B1 cDNA (SP1 5′‐GGCTTCCCAGATTTAGTGAC‐3′ and SP2 5′‐GTGACACAACAAGAACAACTGCG‐3′).

## Results

### The pollen coat of *Brassica* contains polymorphic PCP‐B class cysteine‐rich proteins

In a previous study that characterized the SLG‐binding pollen coat protein PCP‐A1 from *Brassica oleracea* (Doughty *et al*., 1998) two polypeptides were found to copurify with PCP‐A1. These were purified to homogeneity and subjected to N‐terminal sequencing. Each shared an identical six amino acid N‐terminal domain and several conserved cysteine residues arranged in a unique pattern with respect to other known pollen coat protein families (Fig. S3). These were subsequently named *Bo*PCP‐B1 and *Bo*PCP‐B2 (for *B. oleracea* pollen coat protein, class B, 1 and 2, respectively). The partial *Bo*PCP‐B polypeptide sequences permitted cloning of their respective full‐length cDNAs by RACE PCR (GenBank accession numbers: *PCP‐B1*, KX099662; *PCP‐B2*, KX099663). *BoPCP‐B1* and *BoPCP‐B2* are predicted to encode proteins of 79 and 84 amino acids, respectively, with both having a putative 25 amino acid secretory signal peptide (Petersen *et al*., 2011) and a conserved pattern of eight cysteine residues in the mature protein. Based on the N‐terminal sequence data, mature *Bo*PCP‐B1 and *Bo*PCP‐B2 are estimated to have M_r_s of 5490 and 6109, respectively. The localization of the *Bo*PCP‐Bs to the pollen coat, together with their broad similarity to other small cysteine‐rich proteins such as PCP‐A1 (Doughty *et al*., 1998) and the pollen self‐incompatibility determinant SCR (Shiba *et al*., 2001), suggested that they could potentially function in the pollen–stigma interaction.

### PCP‐Bs are evolutionarily widespread and have homology to Arabidopsis Embryo Surrounding Factor 1 developmental regulators

**Figure 1:**
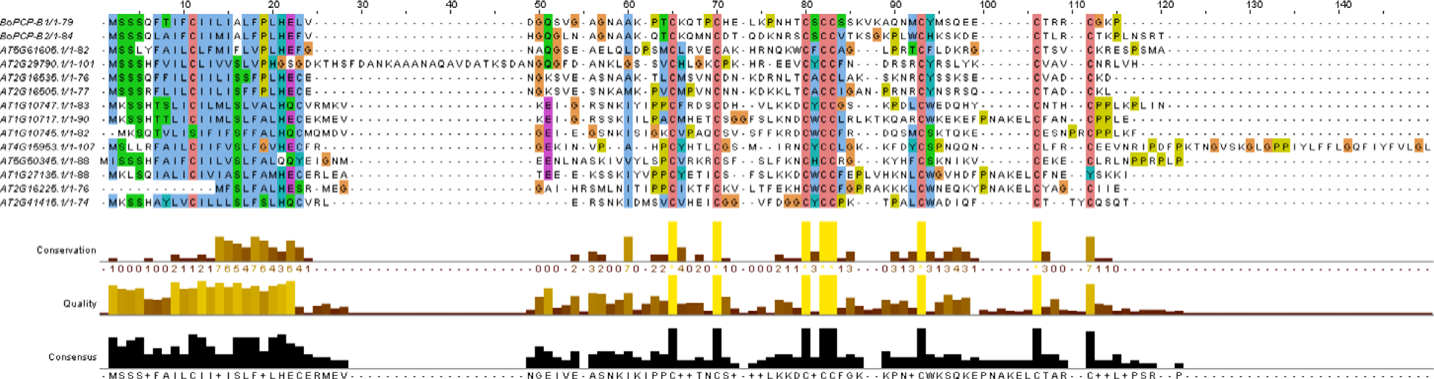
Protein sequence alignment of *Brassica oleracea* PCP‐B1 and PCP‐B2 with all known *Arabidopsis thaliana* (Col‐0) PCP‐B‐like proteins. *At* PCP‐Bs are: *At* PCP‐Bα (At5g61605), *At* PCP‐Bβ (At2g29790), *At* PCP‐Bγ (At2g16535) and *At* PCP‐Bδ (At2g16505). At1g10747, At1g10745 and At1g10717 are ESF1.1, ESF1.2 and ESF1.3, respectively. Sequence conservation, quality and consensus is displayed below. Colour coding follows the default output for Clustal X (http://www.jalview.org/help/html/colourSchemes/clustal.html).

**Figure 2:**
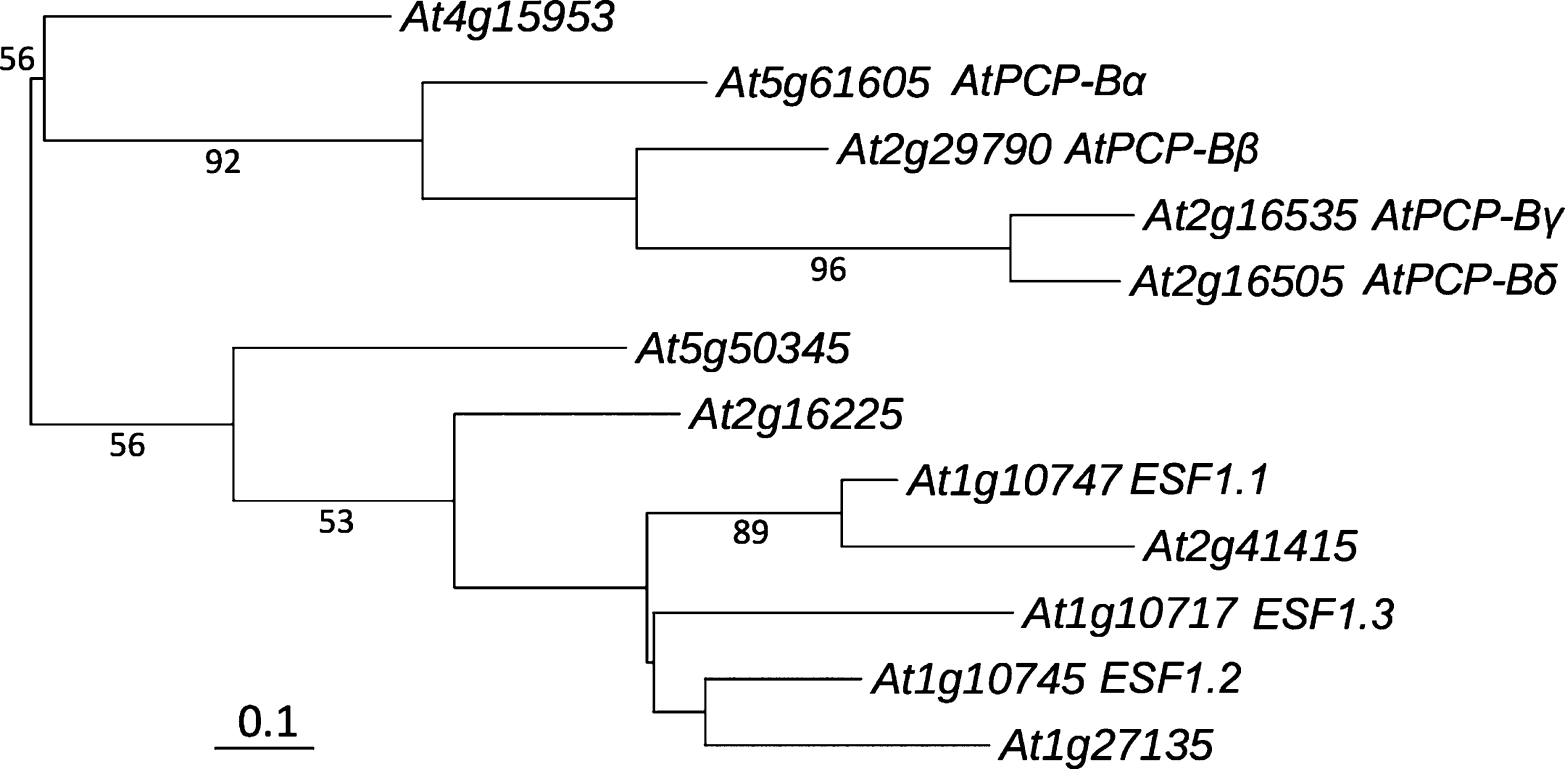
Phylogenetic analysis of 12 *PCP‐B* class genes in *Arabidopsis thaliana* (Col‐0). The maximum‐likelihood tree was constructed by using the nucleotide sequences of predicted mature protein coding regions. Branch lengths are proportional to the bar, defined as 0.1 nucleotide substitutions per codon. The percentage bootstrap values (1000 resamplings) > 50% are shown by interior branches.

**Figure 3:**
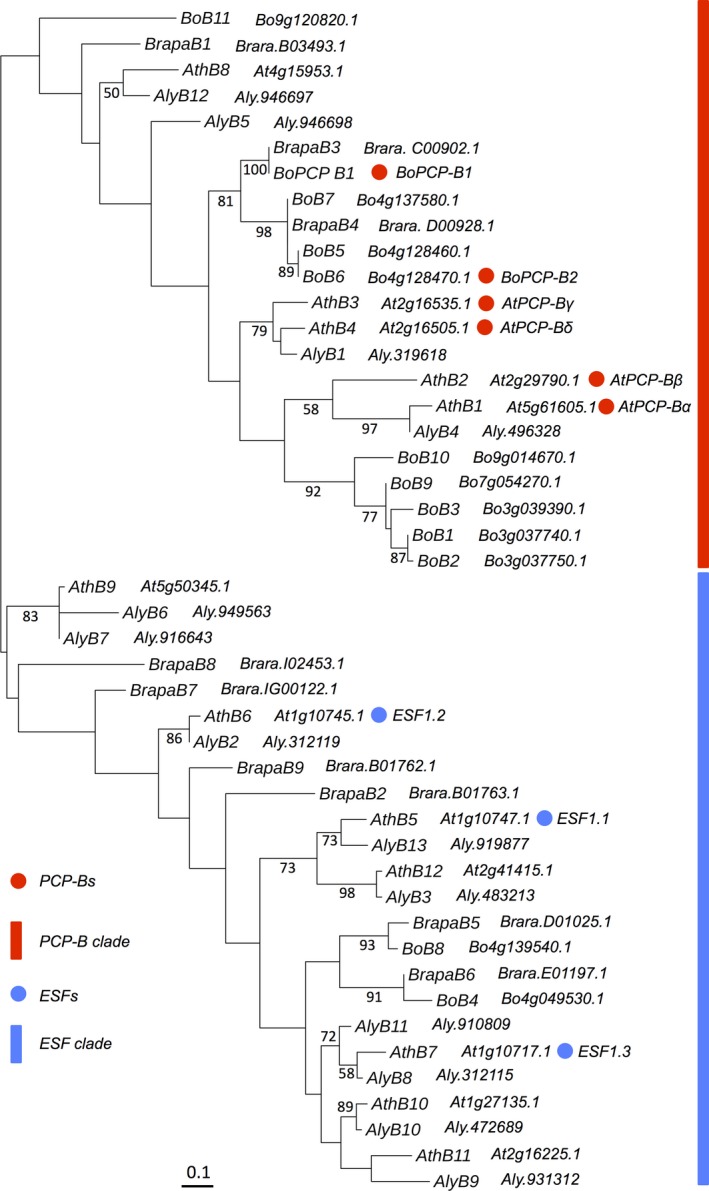
Phylogenetic analysis of 46 *PCP‐B* class genes in *Arabidopsis* and *Brassica*. The maximum‐likelihood tree was constructed using nucleotide sequences of the predicted mature protein coding regions. Bootstrap values (1000 resamplings) > 50% are shown for interior branches. Branch length is scaled to the bar defined as 0.1 nucleotide substitutions per codon. The clades indicated by red and blue bars include *PCP‐Bs* and *ESFs*, respectively. Genes are abbreviated as: *AthB*,* Arabidopsis thaliana PCP‐B‐like*;* AlyB*,* Arabidopsis lyrata PCP‐B‐like*;* BoB*,* Brassica oleracea PCP‐B‐like*;* BrapaB, Brassica rapa PCP‐B‐like*. Gene loci or scaffolds are shown adjacent to gene abbreviations.

**Figure 4:**
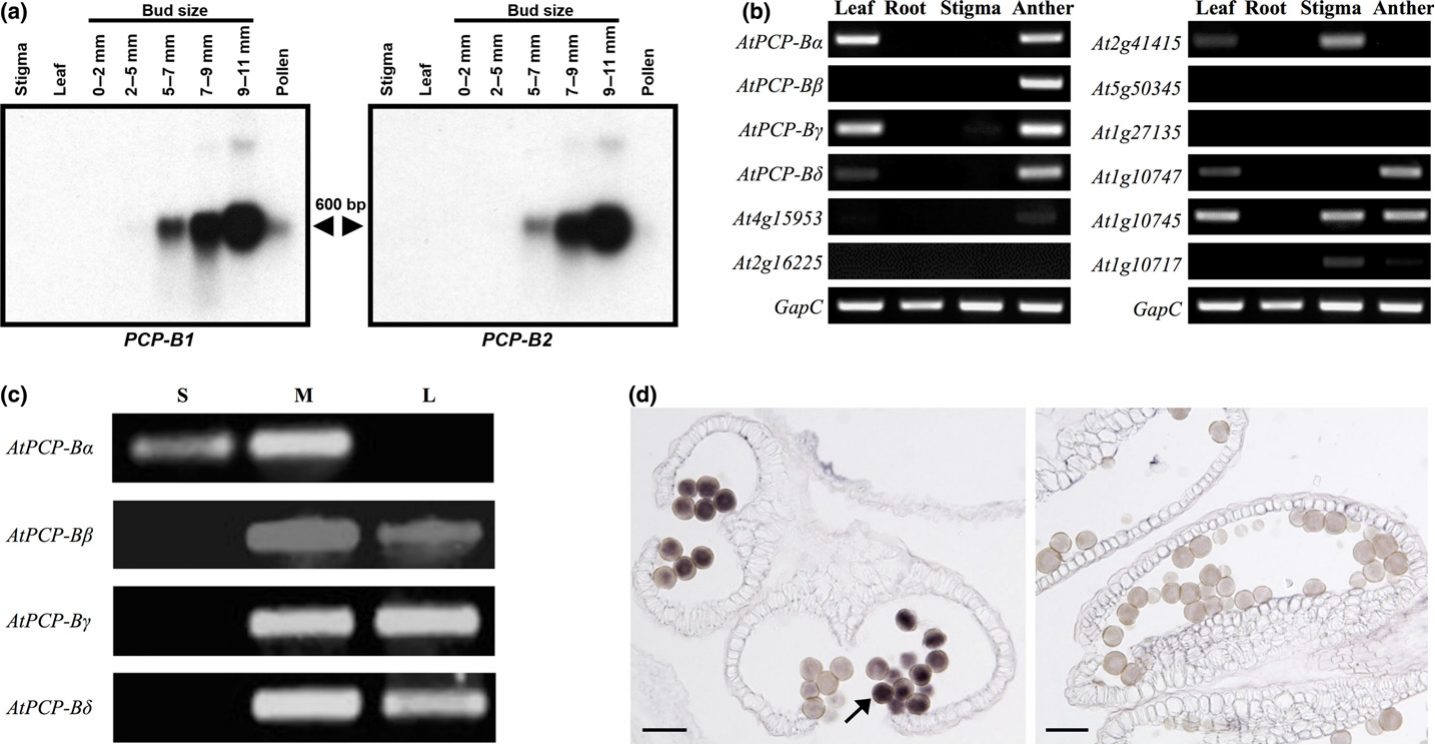
Brassica and Arabidopsis *PCP‐B*s are gametophytically expressed late in pollen development. (a) mRNA gel blot analysis of *Brassica oleracea PCP‐B1* and *PCP‐B2* expression in leaves and reproductive tissue. Anthers from 9 to 11 mm buds have a fully degenerated tapetum and pollen is trinucleate. The arrows indicate the size of the transcript in base pairs. (b) Reverse transcription polymerase chain reaction (RT‐PCR) expression analysis of *AtPCP‐B* and *AtPCP‐B*‐like genes in Arabidopsis leaves, roots, stigmas and anthers (derived from stage 12 buds). *GapC* – cDNA input control for RT‐PCR. (c) *AtPCP‐B* gene expression in flower buds through development (stages 10–12). S, small (< 1 mm; uninucleate microspores); M, medium (*c*. 1–1.5 mm; binucleate pollen); L, large (> 1.5 mm; unopened buds, trinucleate mature pollen). Arabidopsis flower bud stages are as defined by Smyth *et al*. (1990). (d) RNA–RNA *in situ* hybridization study of *AtPCP‐Bβ* expression in *Arabidopsis thaliana* anthers. Left panel: transverse anther section treated with an antisense (+ve) *AtPCP‐Bβ* DIG‐labelled riboprobe, a clear signal (arrow) is observed within the majority of pollen grains. Right panel: longitudinal anther section treated with a control ‘sense’ (−ve) riboprobe with no signal being detectable in pollen grains. Bars, 20 μm.

In order to facilitate subsequent functional analyses of PCP‐B class pollen coat proteins putative homologues were identified in the model plant Arabidopsis. BLAST searching of the Arabidopsis genome using *Bo*PCP‐B sequences revealed the presence of twelve *PCP‐B*‐like genes. All sequences were predicted to encode small, typically basic secreted proteins that shared a common cysteine pattern of seven or eight cysteines in the mature polypeptide (Fig. 1). Importantly, three members of this Arabidopsis gene family (*At1g10747*,* At1g10745* and *At1g10717*) encode the central cell‐derived Embryo Surrounding Factor 1 (ESF1) signalling proteins known to shape early embryo development and patterning (Costa *et al*., 2014). A phylogenetic analysis of mature protein‐encoding gene regions, based on prior alignment of amino acid sequences, revealed that the *AtPCP‐B‐*like sequences fall into two distinct clades (Fig. 2). One clade includes ESF1‐encoding genes clustering into a group of five sequences. Of the other clade four of the sequences fall into a cluster which, following wider phylogenetic analysis across the *Brassica* and *Arabidopsis* genera, placed them in a clade that included genes encoding pollen coat‐derived *Bo*PCP‐Bs (Fig. 3). Expression analyses confirmed these four genes as being largely anther‐specific (Fig. 4). Taken together, these data suggest that the Arabidopsis sequences are likely orthologues of the *B. oleracea PCP‐Bs* and, hence, we named them *AtPCP‐Bα* (*At5g61605*), *AtPCP‐Bβ* (*At2g29790*), *AtPCP‐Bγ* (*At2g16535*) and *AtPCP‐Bδ* (*At2g16505*).

Iterative BLAST searches *Arabidopsis* and *Brassica* genera identified 46 PCP‐B‐like sequences in total across four species. Phylogenetic analysis of these protein sequences revealed not only the high degree of polymorphism across the family, but also the presence of two distinct groupings of sequences, the *PCP‐B* and *ESF1*‐containing clades (Fig. 3). These groupings may reflect functional specialization into seed and pollination‐specific roles. Wider phylogenetic Blast analyses across all known plant lineages identified 282 predicted PCP‐B‐like protein sequences in seven angiosperm families (36 species in total, Fig. S4; Table S2). In addition to the Brassicaceae, PCP‐B‐like proteins were found in the Poaceae, Nelumbonaceae, Solanaceae, Malvaceae, Phrymaceae and Pedaliaceae. Thus, the PCP‐Bs are members of a wider family of highly polymorphic, though structurally related proteins, having an ancient evolutionary origin that predates the split between monocot and eudicot lineages.

### Arabidopsis and *Brassica oleracea PCP‐B* genes are expressed in maturing pollen

RNA gel‐blot analysis for the *Brassica oleracea PCP‐B1* and *PCP‐B2* genes indicated high levels of expression in anthers (Fig. 4a). Transcripts were first detected at low levels in anthers derived from 5 to 7 mm flower buds reaching a maximum by the 9–11 mm bud stage by which time pollen is tricellular and tapetal cells that line the anther locule are fully degraded (Doughty *et al*., 1998). This late pattern of expression in anther development infers that *Bo*PCP‐Bs are likely to be gametophytically derived rather than being products of the tapetum. No expression was detected in leaves and stigmas, and only very low transcript levels were detected in mature pollen. This expression pattern exactly mirrors that of the pollen coat protein gene *PCP‐A1* (Doughty *et al*., 1998). In order to determine which of the twelve Arabidopsis *PCP‐B‐*like genes were likely orthologues of the *B. oleracea PCP‐B* sequences, expression analysis was carried out by RT‐PCR (Fig. 4b). Six of the genes were found to be expressed in stage 12 anthers though two of these, At1g10747 and At1g10745, have previously been characterized as central cell‐derived ESF1 signalling proteins involved in embryo patterning (Costa *et al*., 2014). The remaining four anther‐expressed genes (*At5g61605*,* At2g29790*,* At2g16535* and *At2g16505*,* AtPCP‐Bα* to *δ*, respectively) were found to share a similar temporal expression pattern to the *B. oleracea PCP‐B*s (Fig. 4a,c). In addition, RNA–RNA *in situ* hybridization for *AtPCP‐Bβ* (Figs 4d, S5) and *AtPCP‐Bγ* and promoter‐GUS fusions for *AtPCP‐Bα* and *AtPCP‐Bδ* (Fig. S6) confirmed high‐level expression in pollen, further validating the status of this group as pollen coat protein‐encoding genes. Taken together with the phylogenetic analysis that placed these four genes in the same clade as *BoPCP‐B*s, it is likely that *AtPCP‐Bα*,* AtPCP‐Bβ, AtPCP‐Bγ* and *AtPCP‐Bδ* are orthologous to the *BoPCPs*.

### Pollen hydration is impaired in *pcp‐b* mutants

**Figure 5:**
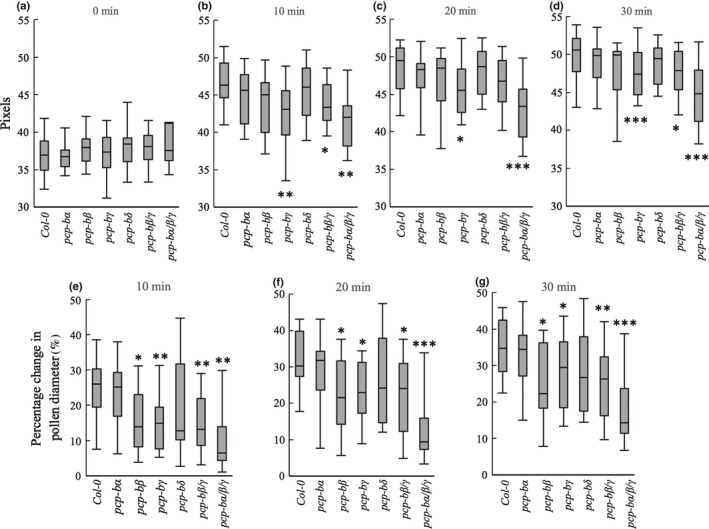
Mutations in *Arabidopsis thaliana PCP‐B* genes result in altered pollen hydration profiles. Box plots depict the 25% quartile, median, 75% quartile and full range of values. (a–d) Pollen diameter distributions at 0, 10, 20 and 30 min following pollination; 1.8 pixels = 1 μm. *, *P *<* *0.05; **, *P *<* *0.001; ***, *P *<* *0.0005 (Welsh's *t*‐test). Sample sizes: Col‐0 (wild‐type), 16; *pcp‐bα*,* pcp‐bβ*,* pcp‐bγ*,* pcp‐bδ*, 15; *pcp‐bα*/*β*, 16; *pcp‐bα*/*β*/*γ*, 15. (e–g) Pollen hydration is represented as percentage change in pollen diameter relative to diameter at 0 min (pollen diameter at initial contact with stigma) – distributions shown are for 10, 20 and 30 min post‐pollination. *, *P *<* *0.05; **, *P *<* *0.005; ***, *P *<* *0.000005 (Welsh's *t*‐test). Sample sizes: Col‐0 (wild‐type), 16; *pcp‐bα*,* pcp‐bβ*,* pcp‐bγ*,* pcp‐bδ*, 15; *pcp‐bα*/*β*, 16; *pcp‐bα*/*β*/*γ*, 15.

In order to investigate the effects of *AtPCP‐B* gene mutations on early stages of the pollen–stigma interaction in *Arabidopsis thaliana*,* in vivo* pollen hydration assays were carried out by pollinating stigmas of the male sterile A9‐barnase WT line with pollen grains derived from WT and *pcp‐b* plants. Four T‐DNA insertion lines were identified as mutant alleles of *AtPCP‐Bα*,* β*,* γ* and *δ* (Fig. S1) with *PCP‐B* transcripts being undetectable in anthers for *pcp‐bα‐1*,* pcp‐bβ‐1* and *pcp‐bγ‐*1. PCP*‐Bδ* expression was found to be substantially downregulated, where the T‐DNA insertion was located in the promoter region of the gene (Figs S1, S2). No obvious vegetative or reproductive morphological abnormalities were observed in any of the lines. Each individual *pcp‐b* line, a double mutant (*pcp‐bβ*/*γ*) and a triple mutant (*pcp‐bα*/*β*/*γ*) were assessed utilizing the pollen hydration assay. A quadruple mutant could not be generated due to the close genetic linkage of *PCP‐Bγ* and *PCP‐Bδ* (*c*. 9 kb apart). Pollen equatorial diameter was recorded for 30 min following placement of pollen on stigmas. Four time points (0, 10, 20 and 30 min) were selected for analysis of the difference of pollen hydration between WT and mutant lines. In addition, the rate of pollen hydration was assessed for each of the three 10‐min periods following pollination. On initiation of pollination (0 min) no significant difference was found between the diameters of pollen derived from WT and mutant lines (Fig. 5a). However, at subsequent time points *pcp‐bγ* and *pcp‐bα*/*β*/*γ* pollen grains were significantly less hydrated than WT pollen (Fig. 5b–d). Assessment of pollen hydration as percentage change in diameter (from time point zero) demonstrated that the degree of pollen hydration was significantly lower in the *pcp‐bβ, pcp‐bγ*,* pcp‐bβ*/*γ* and *pcp‐bα*/*β*/*γ* mutant lines compared with WT at each time point (Fig. 5e–g). Despite there being no statistically significant difference in pollen hydration for *pcp‐bα* and *pcp‐bδ* compared with WT, median pollen diameters, hydration percentage and overall ranges in the data suggested that *pcp‐bα* and *pcp‐bδ* mutations also negatively impact on pollen hydration (Fig. 5e–g).

**Figure 6:**
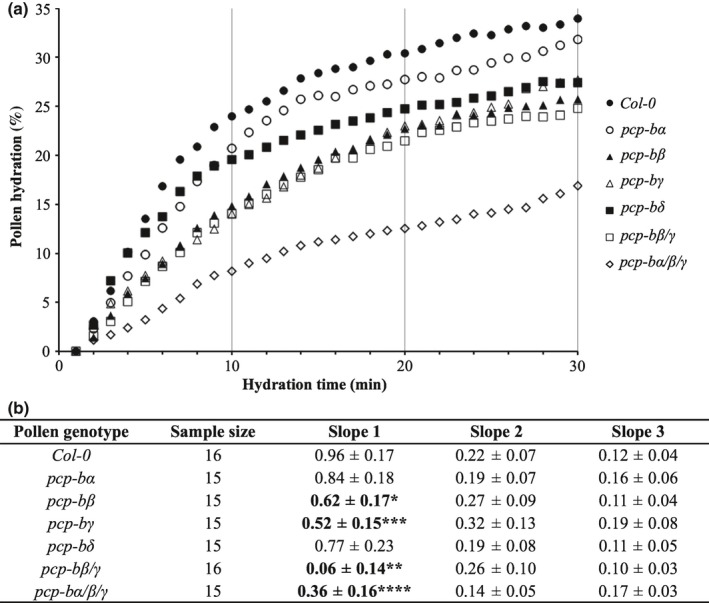
Rate of pollen hydration is severely decreased in *Arabidopsis thaliana pcp‐b* triple mutants. (a) Curves of mean pollen hydration (% hydration is percentage change in pollen diameter). The vertical lines demark each 10‐min period over which slopes were calculated. (b) Rate of change in pollen diameter during the first three 10‐min periods of pollination. Average slopes ± confidence intervals were produced by linear regression. *, *P *<* *0.05; **, *P *<* *0.005; ***, *P *<* *0.001; ****, *P *<* *0.0005 (Welsh's *t*‐test).

We extended the analysis to determine the rate of pollen hydration on stigmas for mutant and WT pollen. Slopes were produced by linear regression based on pollen grain diameter during each 10‐min period following pollination. During the first 10‐min period WT pollen hydrates rapidly with this rate decreasing dramatically during the second and third 10‐min periods. A similar overall pattern was observed for all *pcp‐b* mutant lines (Fig. 6a). During the first 10‐min period of pollination, the hydration rate of WT pollen was significantly higher than that for *pcp‐bβ* and *pcp‐bγ*. The *pcp‐bβ*/*γ* double mutant also demonstrated a significantly affected hydration rate though this was not greater than either single mutant. However, introduction of *pcp‐bα* into the *pcp‐bβ*/*γ* line creating the *pcp‐bα*/*β*/*γ* triple mutant had a dramatic effect on pollen hydration. *pcp‐bα*/*β*/*γ* pollen hydrated at a substantially lower rate than either WT or pollen from the *pcp‐bβ*/*γ* double‐mutant line (Fig. 6) despite the fact that the *pcp‐bα* mutant had little discernible effect on pollen hydration in isolation. Hydration rates for the second and third 10‐min periods were not significantly different to WT for any of the mutant lines although the final extent of hydration was clearly lower for all lines with the exception of *pcp‐bα*.

In order to determine if the pollen hydration defect resulted from an inherent inability of mutant pollen grains to absorb water rather than a defect in the pollen–stigma interaction, an *in vitro* pollen hydration assay was carried out. Using a humid chamber (providing 100% relative humidity) hydration of WT and *pcp‐bα*/*β*/*γ* pollen was compared over a 30‐min period and their hydration characteristics were found to be indistinguishable (Fig. S7). Interestingly, comparison of WT pollen hydration on stigmas and in the humid chamber indicated that pollen hydrates more rapidly, and attains a greater degree of hydration on stigmas (Figs 5b–d, S7b–d). These data demonstrate that the stigma is essential for rapid pollen hydration, and importantly, the absence of PCP‐B protein from the pollen coat does not impair the biophysical ability of pollen to acquire water.

### Pollen adhesion is reduced in *pcp‐b* mutants

**Figure 7:**
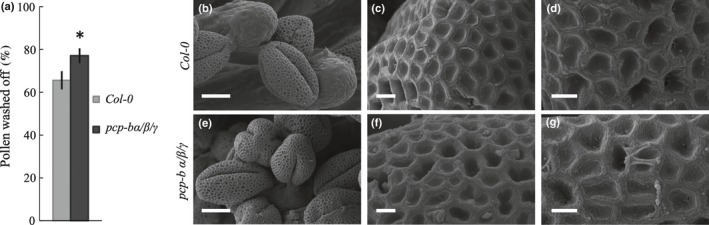
Pollen morphology is unaffected in *Arabidopsis thaliana pcp‐bα*/*β*/*γ* pollen grains and pollen–stigma adhesion is weakened. (a) The mean percentage of wild‐type (Col‐0) and *pcp‐bα*/*β*/*γ* triple mutant pollen washed off WT stigmas in an adhesion assay 30 min post‐pollination. Error bars represent the confidence interval. Sample sizes: WT stigmas, 64; triple mutant, 50. *, *P *<* *0.001 (Welsh's *t*‐test). (b–g) Scanning electron microscopic (SEM) analysis of exine and pollen coat morphology in (b–d) WT and (e–g) *pcp‐bα*/*β*/*γ* triple mutant plants. Bars: (b, e), 10 μm; (c, d, f, g) 1 μm.

In order to further characterize the phenotype of *PCP‐B* mutants, a pollen adhesion assay was devised which tested the ease with which pollen could be washed off the stigma. Significantly higher numbers of pollen grains from the *pcp‐bα*/*β*/*γ* triple mutant (77%) were washed off WT stigmas compared with WT pollen (66%) 30 min post‐pollination (Fig. 7a). However, an EM ultrastructural analysis of the pollen from all *pcp‐b* mutant lines revealed no discernible abnormalities in the characteristics of the pollen grain or pollen coat (Figs 7b–g, S8, S9).

### Initiation of pollen tube growth is delayed in *pcp‐b* mutants

**Figure 8:**
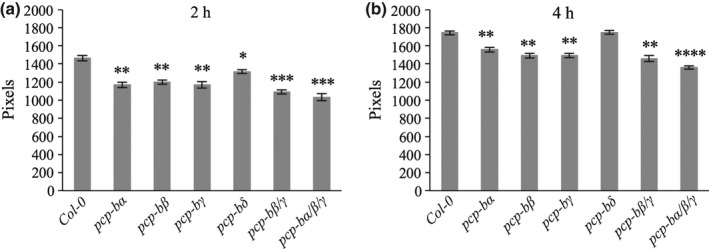
Extent of *in vivo* pollen tube growth is reduced for *pcp‐b* mutants. Distance (in pixels) of pollen tube growth for wild‐type and *pcp‐b* mutants (a) 2 h and (b) 4 h post‐pollination. Pollen was applied to stigmas of the *Arabidopsis thaliana* Col‐0 A9‐barnase male sterile line. Error bars represent ± SD. Sample sizes: 4. *, *P *<* *0.001; **, *P *<* *0.0001; ***, *P *<* *0.00001; ****, *P *<* *0.000001 (Welsh's *t*‐test); 1 pixel = 0.625 μm.

In order to determine if the early stages of pollen tube growth were affected by the delay in pollen hydration observed for *pcp‐b* mutants, *in vivo* pollen tube lengths were estimated. After 2 h WT pollen produced significantly longer tubes than pollen derived from all *pcp‐b* mutant lines (Figs 8a, S10) with this effect being largely maintained 4 h post‐pollination (Fig. 8b). This result is consistent with data collected from the pollen hydration assay where most mutants displayed impairment to the degree and rate of hydration which in turn would likely cause a delay in pollen tube emergence. Despite the observed post‐pollination defects amongst the *pcp‐b* mutants, there was no significant difference in seed set following self‐pollinations compared with WT plants (Table S3), indicating that PCP‐B protein function is likely restricted to very early post‐pollination events.

### Structural prediction of AtPCP‐Bs

**Figure 9:**
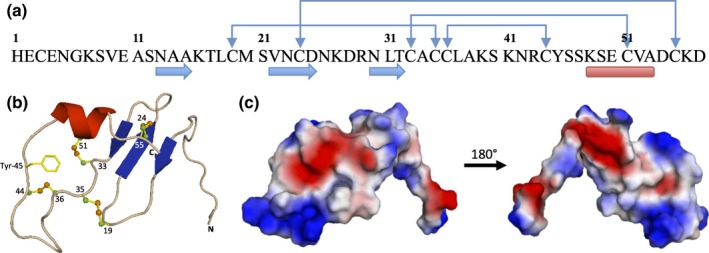
AtPCP‐Bγ structure prediction by SWISS‐MODEL. (a) Amino acid sequence of PCP‐Bγ. Connection arrows, disulphide bonds; blue arrows, beta strands; red bar, alpha helix. (b) Cartoon model of predicted structure of AtPCP‐Bγ with indicated disulphide bonds and Tyrosine residue. (c) Distribution of electrostatic potential on AtPCP‐Bγ surface based on the predicted structure. Blue, positive; red, negative; white, hydrophobic residues.

Our analyses have revealed the presence of PCP‐B‐like proteins in a wide range of angiosperm lineages with all sequences sharing the characteristic motif of eight cysteine residues in the mature polypeptide (Fig. 1). Costa *et al*. (2014) recently resolved the structure of the PCP‐B‐like protein ESF1.3 by nuclear magnetic resonance (NMR) and this made it possible to generate 3D structural predictions for the *At*PCP‐Bs by homologous alignment (Figs 9, S11, S12). All resulting models were statistically well‐supported (Table S4).

Based on the predicted 3D structure *At*PCP‐Bs likely share the same intramolecular disulphide bonding pattern as ESF1.3 (Figs 9a,b, S12) with all possessing a conserved cysteine‐stabilized motif consisting of an α‐helix and three‐stranded antiparallel beta‐sheet. In addition all *At*PCP‐Bs have a conserved aromatic residue (Tyr‐45 in *At*PCP‐Bγ) that is also present in ESF1.3 (Trp‐48) and other PCP‐B‐like proteins in *Arabidopsis thaliana* (Fig. 1). The surface electrostatic potential distribution for *At*PCP‐Bγ (Fig. 9c) is characterized by both positively and negatively charged domains with a prominent positively charged extended loop held between Cys‐36 and Cys‐44 which lies in close proximity to the conserved aromatic residue (Tyr‐45). These features are broadly shared between all four *At*PCP‐Bs (Figs 9, S12).

## Discussion

Compatible pollination is a highly regulated process that requires a suite of complementary pollen and pistil factors that act from the moment of pollen contact through to successful fusion of gametes (Edlund *et al*., 2004; Hiscock & Allen, 2008). One of the earliest events in the establishment of compatibility amongst species that possess dry stigmas, such as *Arabidopsis thaliana*, is the ability for pollen to gain access to stigmatic water (Elleman *et al*., 1992; Safavian & Goring, 2013). This reproductive ‘checkpoint’ requires activation of a basal stigmatic compatibility system by factor(s) that must be derived from pollen (Safavian & Goring, 2013). Our investigations reported here into small pollen coating‐borne cysteine‐rich proteins point to an important role for the PCP‐Bs in these earliest stages of pollen–pistil compatibility in Arabidopsis, as plants carrying mutations in *PCP‐B* genes are impaired in pollen hydration. Importantly, PCP‐Bs bear many hallmarks of intercellular signalling ligands and thus are likely to be a central component of a pollen molecular ‘signature’ that defines compatibility.

PCP‐Bs are structurally related proteins that have an ancient evolutionary origin, being widespread amongst angiosperm taxa. Our phylogenetic analysis of 46 gene sequences encoding PCP‐B‐like proteins in *Arabidopsis* and *Brassica* (Fig. 3) reveals an evolutionary history featuring frequent gene duplication events and rapid sequence divergence around their conserved cysteine motif. These features are typical for gene families associated with reproduction and importantly can contribute to reproductive isolation and speciation (Swanson & Vacquier, 2002; Clark *et al*., 2006; Cui *et al*., 2015). Interestingly the *PCP‐Bs* investigated here were found to be closely related to the *ESF1*s that encode embryo developmental regulators (Costa *et al*., 2014) and these sequences clustered in distinct phylogenetic clades, underlining their functional specialization (Figs 2, 3). Some *AtPCP‐B* family members were more similar to genes in the closely related species *A. lyrata* suggesting that these have retained a specific function that predates speciation. For example, *AtPCP‐Bα* and *AtPCP‐Bγ* are more closely related to the *A. lyrata B4* and *B1,* respectively, than to other *AtPCP‐Bs* (Fig. 3). Whereas putative *Brassica* orthologues were found in discrete clades more distant from the *Arabidopsis PCP‐Bs* and could point to divergence of recognition factors required for compatibility. Species‐specific functionalization of plant reproductive proteins that contribute to reproductive isolation have been documented for the pollen tube attractant LURE proteins secreted by egg‐accompanying synergid cells of the embryo sac (Takeuchi & Higashiyama, 2012). Heterologous expression of an *A. thaliana* LURE protein in *Torenia fournieri* synergid cells enabled *A. thaliana* pollen tubes to successfully locate and enter the embryo sac of this species. LURES are defensin‐like CRPs and, in common with the PCP‐B class proteins, are small secreted proteins that are encoded by a rapidly evolving gene family. It is thus tempting to speculate that PCP‐Bs not only regulate aspects of compatibility but may also contribute to reproductive barriers within the Brassicaceae.

Our mutational study revealed that absence of *At*PCP‐Bs from the pollen coat caused a series of interlinked phenotypes resulting from a primary defect in pollen hydration. We ascertained that the *pcp‐b* hydration defect was not caused by gross morphological perturbation of the pollen coat (Figs 7b–g, S8, S9) and that it was only evident during the pollen–stigma interaction, as triple mutant *pcp‐bα*/*β*/*γ* pollen hydrated normally in a humid chamber (Fig. S7). Hydration rate, the degree of hydration and resulting pollen tube lengths were all found to be largely impaired amongst *pcp‐b* single and combined mutants (Figs 5, 6, 8). We consider that the shorter tubes observed in pistils for *pcp‐b* mutants are most likely the result of delayed pollen tube emergence rather than slower tube extension, because tube emergence is largely dependent on the degree of pollen hydration and pollen turgor (Taylor & Hepler, 1997). This inference was supported by the observation that triple mutant pollen adhered significantly less well to stigmatic papillae 30 min post‐pollination (Fig. 7a) – we observed that a significant component of this effect was due to WT pollen tubes initiating stigmatic penetration ahead of *pcp‐b* pollen, thus anchoring them on the stigma, whereas substantially fewer mutant pollen had initiated germination (L. Wang & J. Doughty, unpublished).

Comparison of the severity of the hydration defects between single and combined mutants revealed evidence of complex combinatorial effects of PCP‐Bs in the pollen–stigma interaction. Out of the single mutant lines, *pcp‐bγ* presented the most statistically robust hydration defect over the first 10‐min period following pollination, with *pcp‐bβ* having an almost identical hydration profile (Figs 5e, 6). Interestingly, the phenotype of the double *pcp‐bβ*/*γ* mutant was not additive, but when combined with the *pcp‐bα* mutant – which singly had no significant phenotype – pollen hydration was reduced dramatically (Figs 5e–g, 6). The contrasting combinatorial effects of these mutants suggests that PCP‐Bα may be acting as a ligand to activate a different stigmatic hydration effector target to that of PCP‐Bβ and PCP‐Bγ, or that PCP‐Bα acts to enhance activation of a putative stigmatic target working synergistically with other PCP‐Bs. Similar complexity has been reported for synergid LURE proteins in *T*. *fournieri* and Arabidopsis where it seems likely that multiple LUREs work together, probably through different pollen tube receptors, to ensure appropriate pollen tube guidance to the embryo sac (Okuda *et al*., 2009; Takeuchi & Higashiyama, 2012, 2016; Wang *et al*., 2016). The severity of the triple *pcp‐bα*/*β*/*γ* mutant reduced the degree and rate of pollen hydration to almost one third that of WT and due to the close genetic linkage of *PCP‐Bδ* to *PCP‐Bγ* (< 10 kb) we were unable to recover and test the effect of a *pcp‐b* quadruple mutant. Thus, it remains to be determined if a complete hydration block could be achieved by abolishing all PCP‐B proteins from the pollen coat.

Given the structural features of *At*PCP‐Bs and their homology to the ESF1 family of secreted developmental regulators we propose that PCP‐Bs act as ligands to either directly or indirectly activate stigmatic targets that mediate transfer of water through the papilla plasma membrane. A substantial body of evidence now points to targeted stigmatic secretion as being a central feature of compatible pollination in both *A. thaliana* and *Brassica* and that the exocyst protein complex is essential to this process (Samuel *et al*., 2009; Safavian & Goring, 2013; Safavian *et al*., 2014, 2015). The exocyst mediates tethering of secretory vesicles to target membranes (Zarsky *et al*., 2013) and stigmas from Arabidopsis that carry mutations in Exo70A1, a key linker component of the exocyst‐tethering machinery, have severe pollen hydration defects. Targeted secretion likely delivers factors to the plasma membrane adjacent to compatible pollen that mediate water transport. For instance, aquaporins, membrane‐localized water transport proteins (Johanson *et al*., 2001; Quigley *et al*., 2002; Maurel *et al*., 2008), could be deposited at the interface with compatible pollen. A specific role for pollen coat factors triggering such a response is supported by the observation that isolated *B. oleracea* pollen coat appears to evoke a secretory response by stigmatic papillae (Elleman & Dickinson, 1996).

Homology modelling of *At*PCP‐Bs provided strong support for overall structural similarity with ESF1.3 (Figs 9b, S12). As has been determined for ESF1.3 and other plant regulatory peptides it is likely that the disulphide‐stabilized cysteine motif is crucial for protein function of PCP‐Bs (Ohki *et al*., 2011; Costa *et al*., 2014). Intriguingly the *At*PCP‐Bs shared a functionally essential aromatic residue with ESF1.3. Aromatic residues are a conserved feature of many plant regulatory peptides (Cao *et al*., 2008; Okuda *et al*., 2009; Sugano *et al*., 2010; Costa *et al*., 2012; Sprunck *et al*., 2012) and are likely to be important in protein–protein interactions (Simpson *et al*., 2000).

In conclusion, this study shows that *At*PCP‐Bs are important mediators of pollen hydration, a key early ‘checkpoint’ of pollen–stigma compatibility. Their close evolutionary relationship to the ESF1 family of embryo developmental regulators, and their broad similarity to other CRP regulatory proteins strongly suggest that they act through interaction with as yet unknown stigmatic targets to activate the basal compatibility system. In addition, PCP‐B maintenance and diversity within *Arabidopsis* and the Brassicaceae suggest that these proteins have the potential to contribute to prezygotic hybridization barriers.

## Author contributions

J.D. and L.W. planned and designed the research and wrote the manuscript; L.W. was involved with all aspects of the research with L.A.C. and R.J.E. contributing to expression analysis and C.C.P. to the SEM work; R.J.S. and B.Q. assisted with experimental design and critical assessment of the manuscript.

## Supporting information

Please note: Wiley Blackwell are not responsible for the content or functionality of any Supporting Information supplied by the authors. Any queries (other than missing material) should be directed to the *New Phytologist* Central Office.

**Fig. S1** Locations of T‐DNA insertions.
**Fig. S2 **RT‐PCR analysis results of stage 12 anthers in *pcp‐b* mutants.
**Fig. S3 **N‐terminal sequencing of two PCP‐B proteins purified from *Brassica oleracea* pollen coat.
**Fig. S4 **Phylogeny of 282 predicted PCP‐B‐like protein sequences.
**Fig. S5 **RNA–RNA *in situ* hybridization study of *AtPCP‐Bγ* expression in *Arabidopsis thaliana* anthers.
**Fig. S6 **Histochemical staining for GUS activity driven by *AtPCP‐Bα* and *AtPCP‐Bδ* promoters in Arabidopsis tissues.
**Fig. S7 **Pollen hydration profiles of wild‐type and *pcp‐b* triple mutant grains in a humid chamber.
**Fig. S8 **SEM analysis of exine layer and pollen coat morphology.
**Fig. S9 **TEM analysis of exine layer and pollen coat morphology.
**Fig. S10** Comparison of pollen tube growth for wild‐type and *pcp‐b* triple mutant plants.
**Fig. S11 **Homologous alignments of ESF1.3 and AtPCP‐Bs for protein structure predictions.
**Fig. S12 **Predicted protein structure homology models of AtPCP‐Bα, β and δ.
**Table S1** PCR primers used in this study
**Table S2 **Numbers and abbreviations of predicted PCP‐B‐like proteins in species and families
**Table S3 **Average seed count values of Arabidopsis wild‐type and *pcp‐b* mutants
**Table S4 **Statistics for AtPCP‐B protein structural predictions
**Methods S1 **Histochemical staining for β‐glucuronidase activity.
