# Supplementary material for: PCP‐B class pollen coat proteins are key regulators of the hydration checkpoint in Arabidopsis thaliana pollen–stigma interactions
Source: New Phytol. 2016 Sep 6;213(2):764–77. doi: 10.1111/nph.14162 (PMC5215366; doi:10.1111/nph.14162)
Supplement: Supplementary file 1 — Fig. S1 Locations of T‐DNA insertions. Fig. S2 RT‐PCR analysis results of stage 12 anthers in pcp‐b mutants. Fig. S3 N‐terminal sequencing of two PCP‐B proteins purified from Brassica oleracea pollen coat. Fig. S4 Phylogeny of 282 predicted PCP‐B‐like protein sequences. Fig. S5 RNA–RNA in situ hybridization study of AtPCP‐Bγ expression in Arabidopsis thaliana anthers. Fig. S6 Histochemical staining for GUS activity driven by AtPCP‐Bα and AtPCP‐Bδ promoters in Arabidopsis tissues. Fig. S7 Pollen hydration profiles of wild‐type and pcp‐b triple mutant grains in a humid chamber. Fig. S8 SEM analysis of exine layer and pollen coat morphology. Fig. S9 TEM analysis of exine layer and pollen coat morphology. Fig. S10 Comparison of pollen tube growth for wild‐type and pcp‐b triple mutant plants. Fig. S11 Homologous alignments of ESF1.3 and AtPCP‐Bs for protein structure predictions. Fig. S12 Predicted protein structure homology models of AtPCP‐Bα, β and δ. Table S1 PCR primers used in this study Table S2 Numbers and abbreviations of predicted PCP‐B‐like proteins in species and families Table S3 Average seed count values of Arabidopsis wild‐type and pcp‐b mutants Table S4 Statistics for AtPCP‐B protein structural predictions Methods S1 Histochemical staining for β‐glucuronidase activity. [file NPH-213-764-s001.pdf]

New Phytologist

New Phytologist Supporting Information Figs S1-S12, Tables S1-S4 and Methods S1

Article title: PCP-B class pollen coat proteins are key regulators of the hydration checkpoint in *Arabidopsis thaliana* pollen–stigma interactions

Authors: Ludi Wang, Lisa A. Clarke, Russell J. Eason, Christopher C. Parker, Baoxiu Qi, Rod J. Scott and James Doughty

Article acceptance date: 23 July 2016

The following Supporting Information is available for this article:

Fig. S1 Locations of T-DNA insertions.

Fig. S2 RT-PCR analysis results of stage 12 anthers in *pcp-b* mutants.

Fig. S3 N-terminal sequencing of two PCP-B proteins purified from *Brassica oleracea* pollen coat.

Fig. S4 Phylogeny of 282 predicted PCP-B-like protein sequences.

Fig. S5 RNA-RNA *in situ* hybridisation study of *AtPCP-Bγ* expression in *Arabidopsis thaliana* anthers.

Fig. S6 Histochemical staining for GUS activity driven by *AtPCP-Bα* and *AtPCP-Bδ* promoters in Arabidopsis tissues.

Fig. S7 Pollen hydration profiles of wild-type and *pcp-b* triple mutant grains in a humid chamber.

Fig. S8 Scanning electron microscopic analysis of exine layer and pollen coat morphology.

Fig. S9 Transmission electron microscopic analysis of exine layer and pollen coat morphology.

Fig. S10 Comparison of pollen tube growth for wild-type and *pcp-b* triple mutant plants.

Fig. S11 Homologous alignments of ESF1.3 and AtPCP-Bs for protein structure predictions.

Fig. S12 Predicted protein structure homology models of AtPCP-Bα, β and δ.

Table S1 PCR primers used in this study

Table S2 Numbers and abbreviations of predicted PCP-B-like proteins in species and families

Table S3 Average seed count values of Arabidopsis wild-type and *pcp-b* mutants

Table S4 Statistics for AtPCP-B protein structural predictions

Methods S1 Histochemical staining for β-glucuronidase activity.

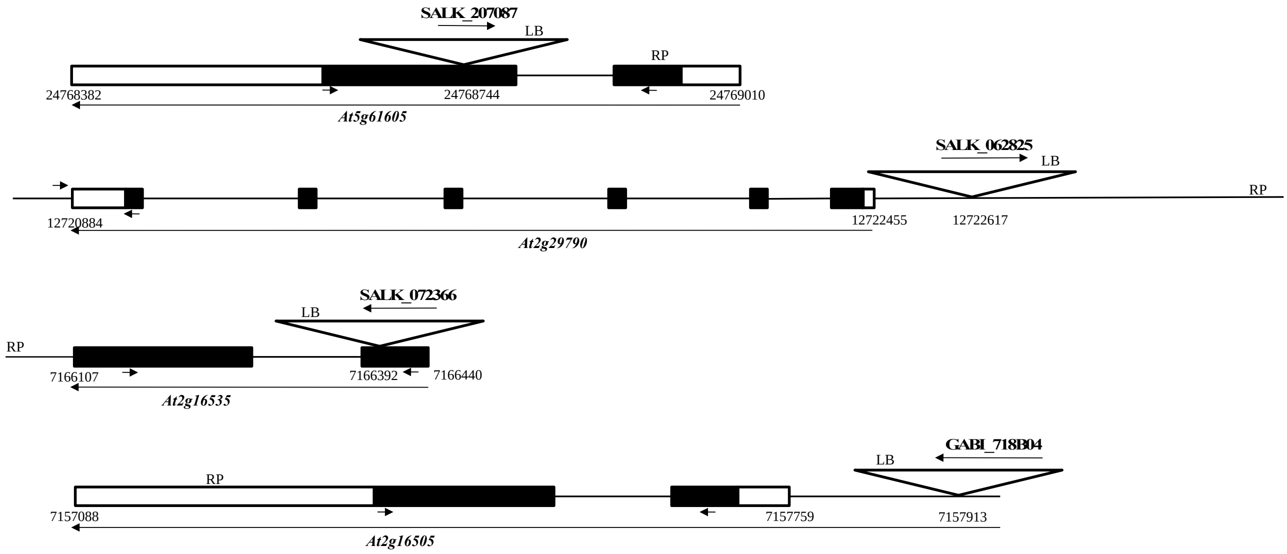

Fig. S1 T-DNA insertion locations of the *pcp-b* mutants. Open boxes, UTR untranscribed regions; closed boxes, exons; horizontal lines, introns; triangles, T-DNA insertion sites. LB and RP indicate the location of primers used in the confirmation of T-DNA insertions. Short arrows indicate the locations of primers used in RT-PCR for the conformation of gene knockouts. *PCP-B* mutant lines are named as follows: SALK_207087, *pcp-bα-1*; SALK_062825, *pcp-bβ-1*; *SALK_072366*, *pcp-bγ-1*; *GABI_718B04*, *pcp-bδ-1*.

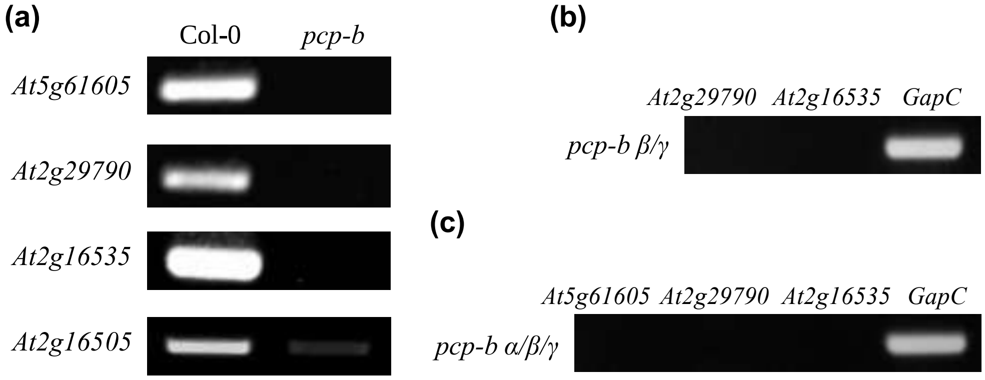

Fig. S2 RT-PCR expression analysis of *PCP-B*s in stage 12 anthers of T-DNA single gene mutant lines (a), the *pcp-b β/γ* double gene mutant line (b) and *pcp-b α/β/γ* triple gene mutant line (c).

PCP-B1: AGNAAKPT**C**KQTP**C**HELKPNHT**C**S**C**
PCP-B2: AGNAAKQT**C**KQMN**C**DTGDKN

Fig. S3 N-terminal sequencing of two PCP-B proteins purified from *Brassica oleracea* pollen coat. The conserved cysteine residues are shown as bold letters. The shared six amino acid N-terminal region is underlined.

New Phytologist

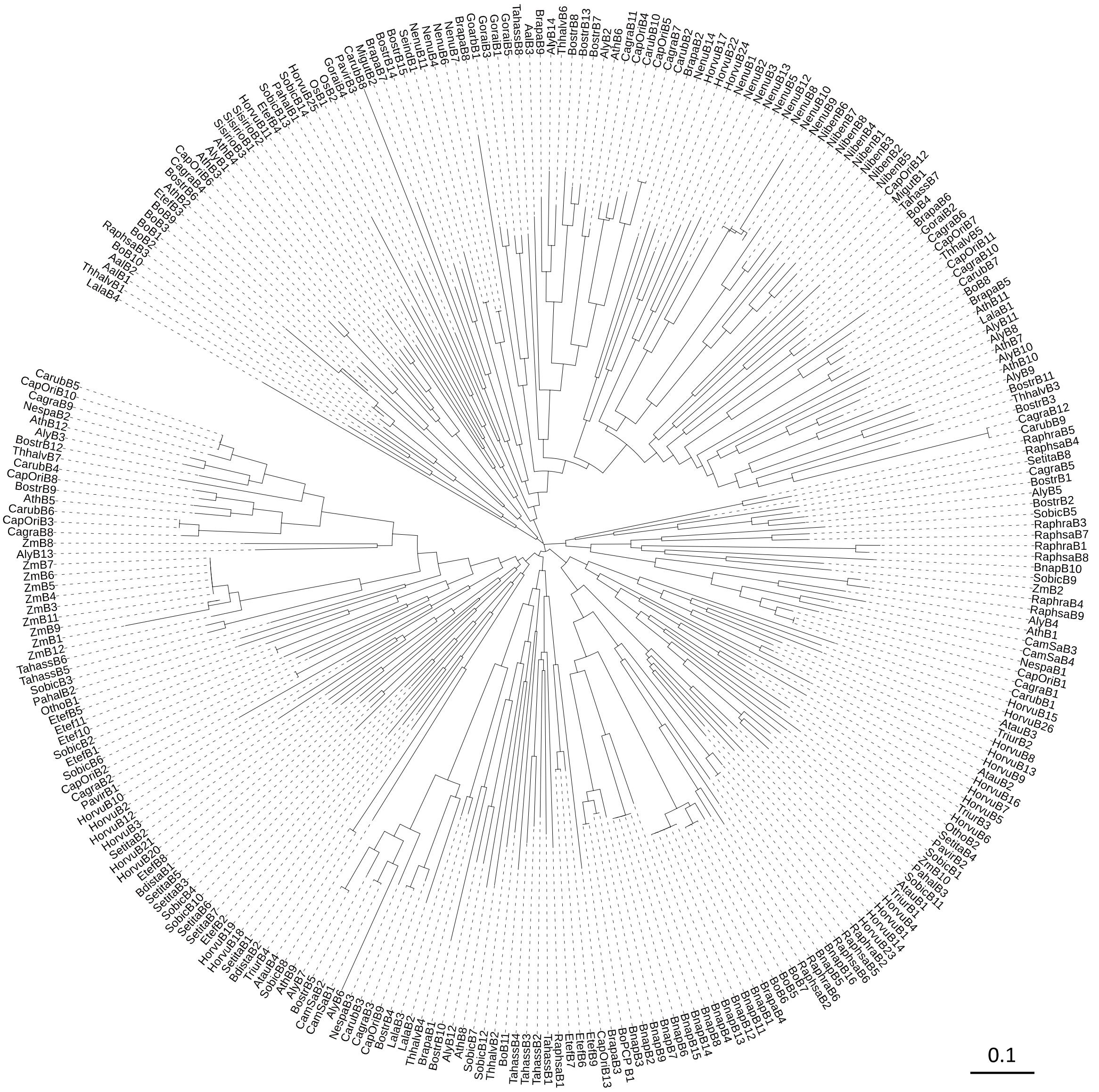

Fig. S4 Phylogeny of PCP-Bs and PCP-B-like proteins in angiosperms. The neighbour-joining tree was constructed using amino acid sequences of the predicted mature proteins. Branch length is scaled to the scale bar defined as 0.1 substitutions per site. Abbreviated gene names can be found in Table S2.

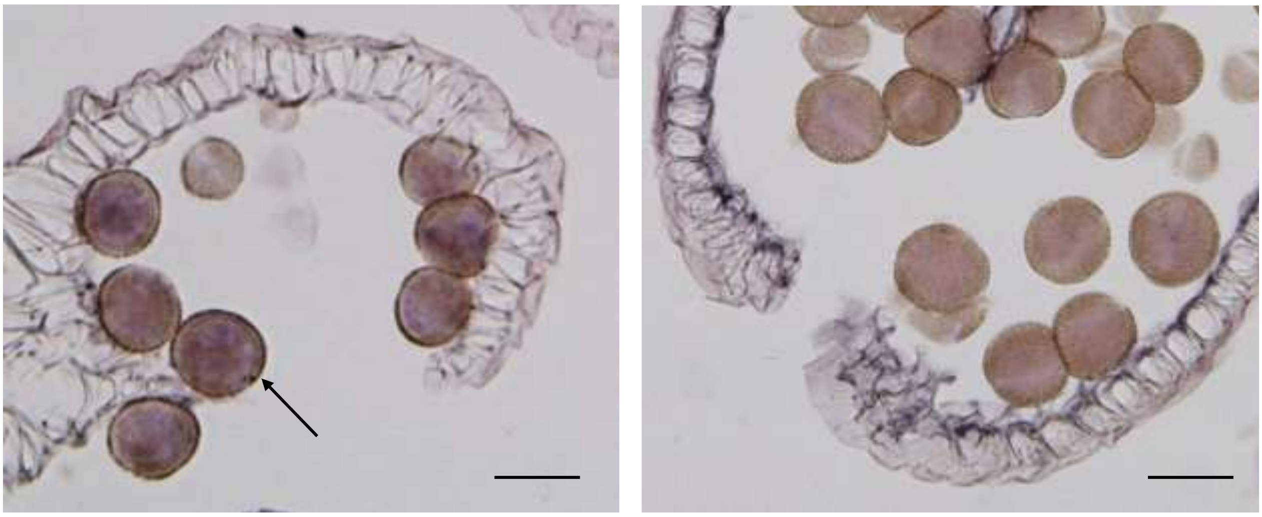

Fig. S5 RNA-RNA *in situ* hybridisation study of *AtPCP-Bγ* expression in *Arabidopsis thaliana* anthers. Left panel: transverse anther section treated with an antisense (+ve) *AtPCP-Bγ* DIG-labelled riboprobe, a clear signal (arrow) is observed within the majority of pollen grains. Right panel: transverse anther section treated with a control ‘sense’ (-ve) riboprobe with no signal being detectable in pollen grains. Bars, 10 μm.

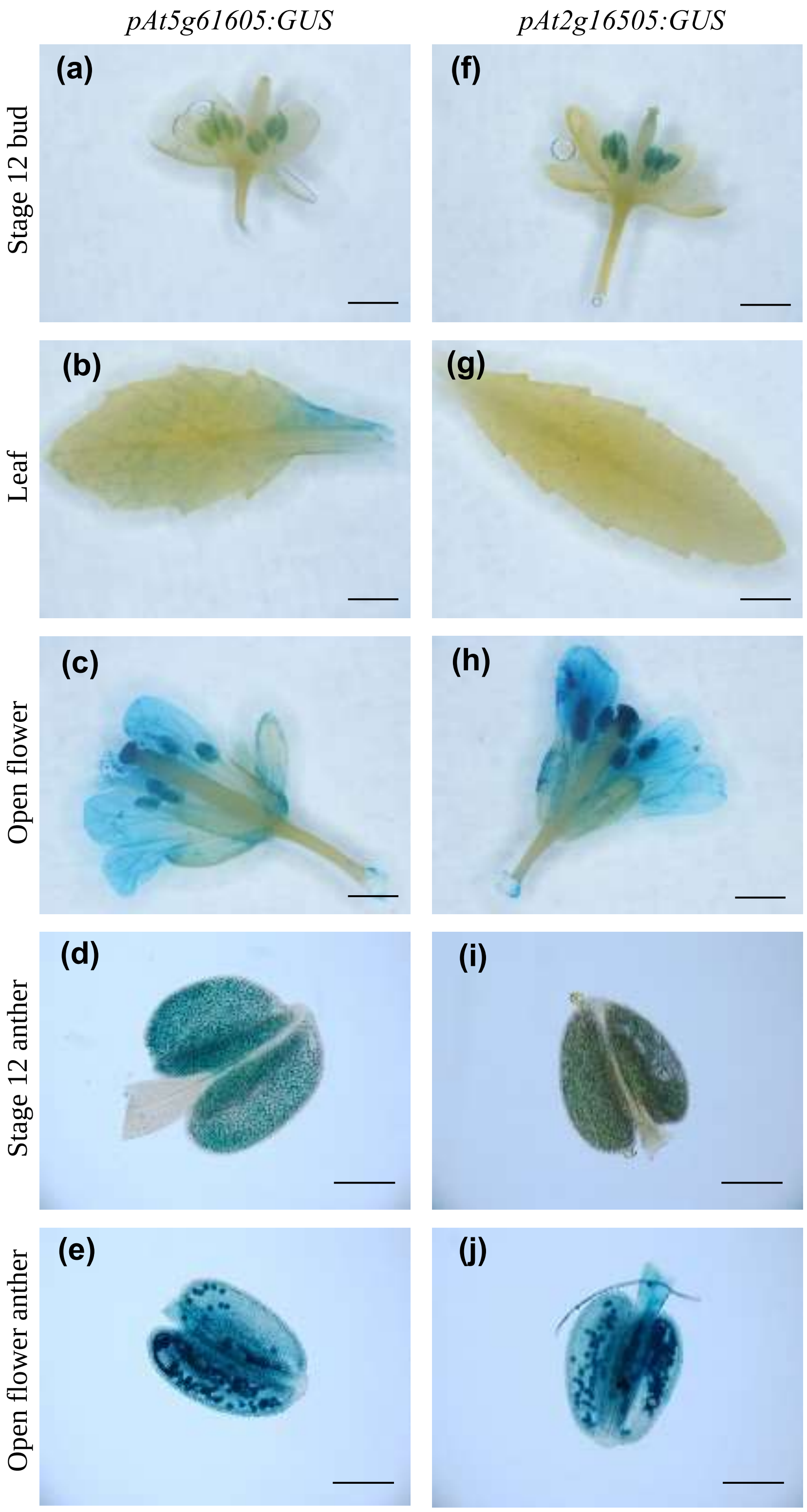

Fig. S6 GUS expression in *Arabidopsis thaliana* tissues driven by (a–e) *AtPCP-Bα* promoter *pAt5g61605* and (f–j) *AtPCP-Bδ* promoter *pAt2g16505*. Bars: (a–c, f–h) 1 mm; (d, e, i, j) 0.2 mm. Signal in stigmatic tissues is the result of pollen deposition.

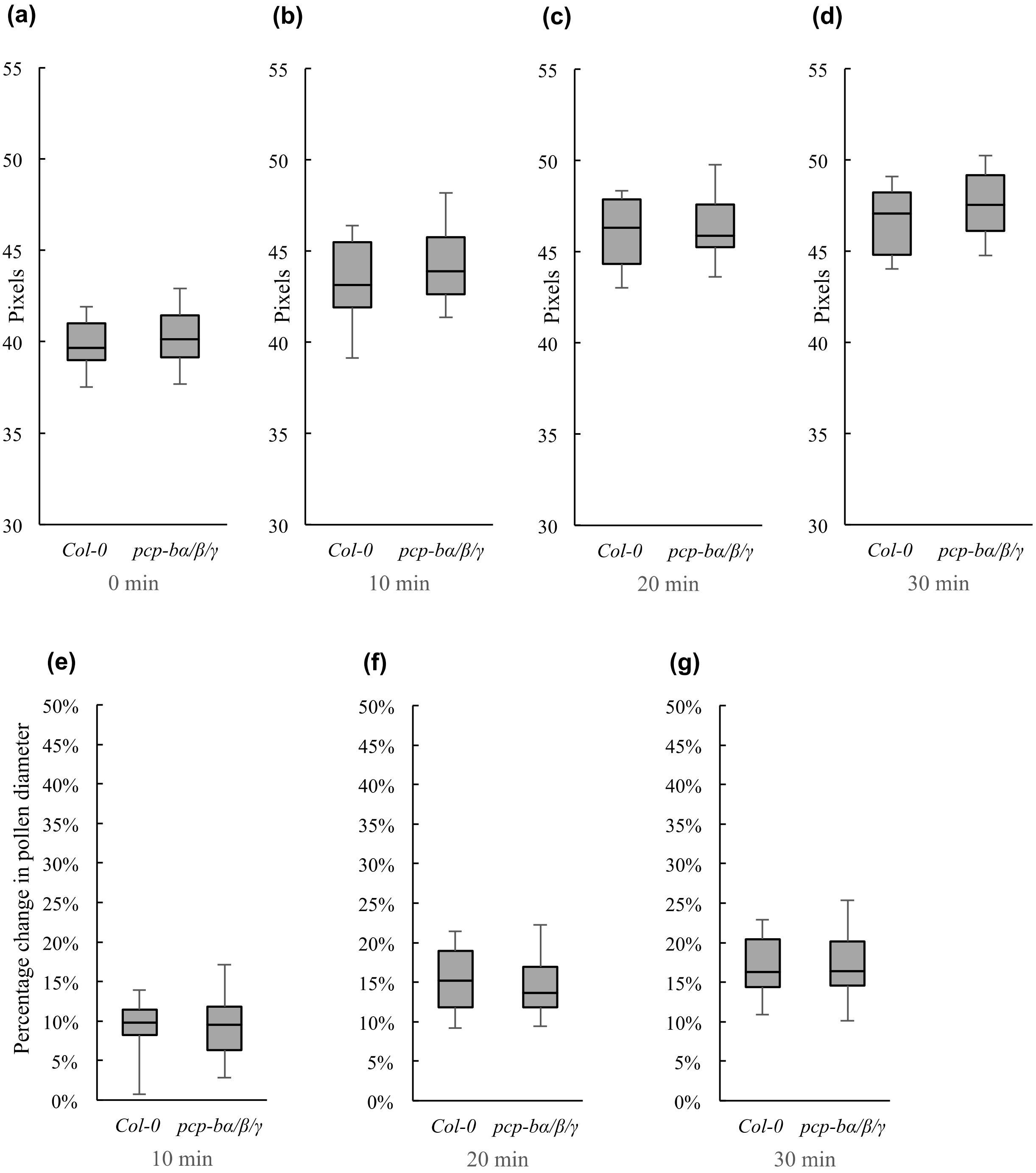

Fig. S7 Pollen hydration profiles of wild-type and *pcp-b* triple mutant pollen in a humid chamber. (a–d) Pollen diameter distributions at 0 min, 10 min, 20 min and 30 min of hydration in a humid chamber. Box plots depict the 25% quartile, median, 75% quartile and full range of values. Sample sizes: 20. (e–g) Distribution of percentage change in pollen diameter at 10 min, 20 min and 30 min of hydration in the humid chamber. Box plots depict the 25% quartile, median, 75% quartile and full range of values. Sample sizes: 20. *P*-values: (a) 0.32; (b) 0.30; (c) 0.79; (d) 0.13; (e) 0.71; (f) 0.46; (g) 0.52 (Welsh's *t*-test). 1.8 pixels = 1 μm.

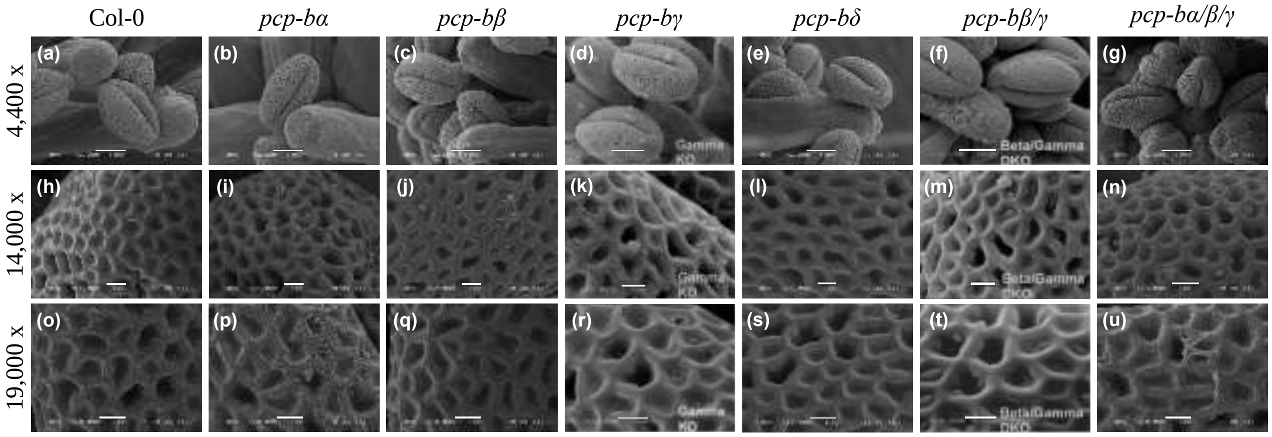

Fig. S8 Scanning electron microscopic (SEM) analysis of exine layer and pollen coat morphology. Bars: (a–g) 10 µm; (h–u) 1 µm. (a, h, o) Wild-type Col-0; (b, i, p) *pcp-bα*; (c, j, q) *pcp-bβ*; (d, k, r) *pcp-bγ*; (e, l, s) *pcp-bδ*; (f, m, t) *pcp-bβ/γ*; (g, n, u) *pcp-bα/β/γ*.

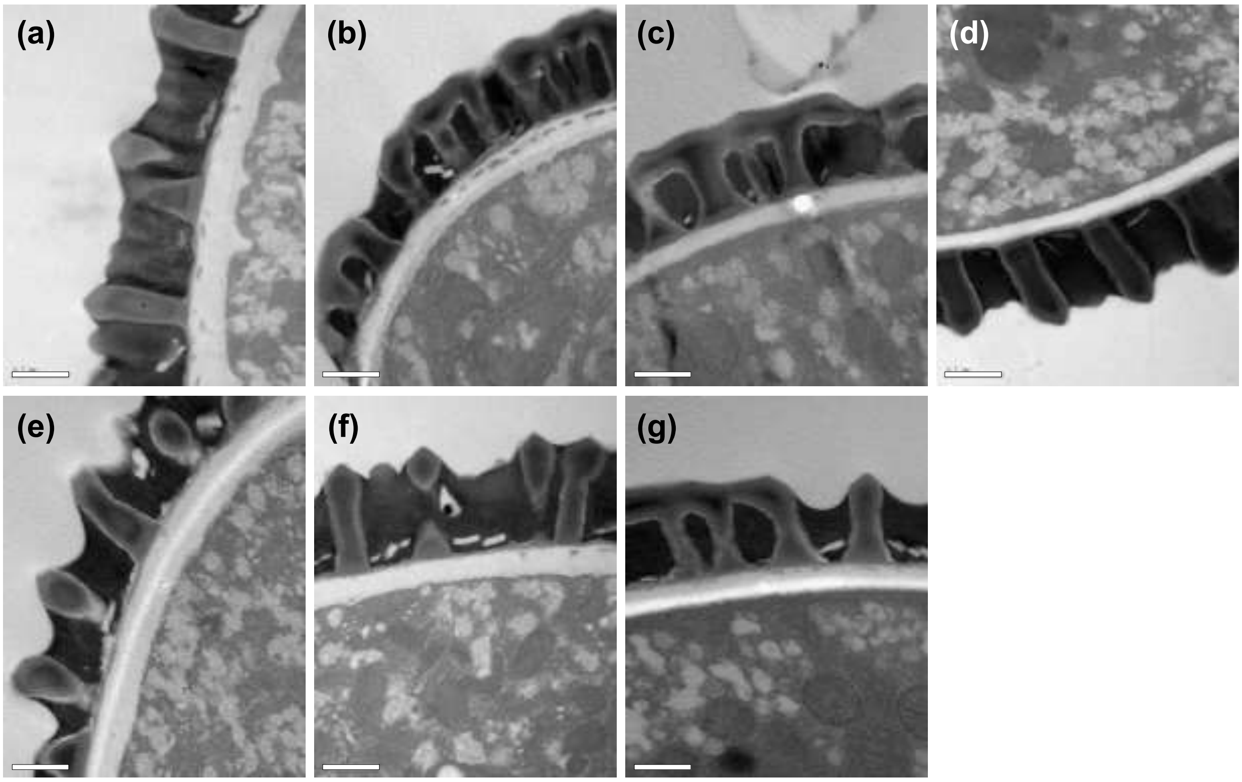

Fig. S9 Transmission electron microscopic (TEM) analysis of exine layer and pollen coat morphology. Bars, 0.5 μm. (a) Wild-type Col-0; (b) *pcp-bα*; (c) *pcp-bβ*; (d) *pcp-bγ*; (e) *pcp-bδ*; (f) *pcp-bβ/γ*; (g) *pcp-bα/β/γ*.

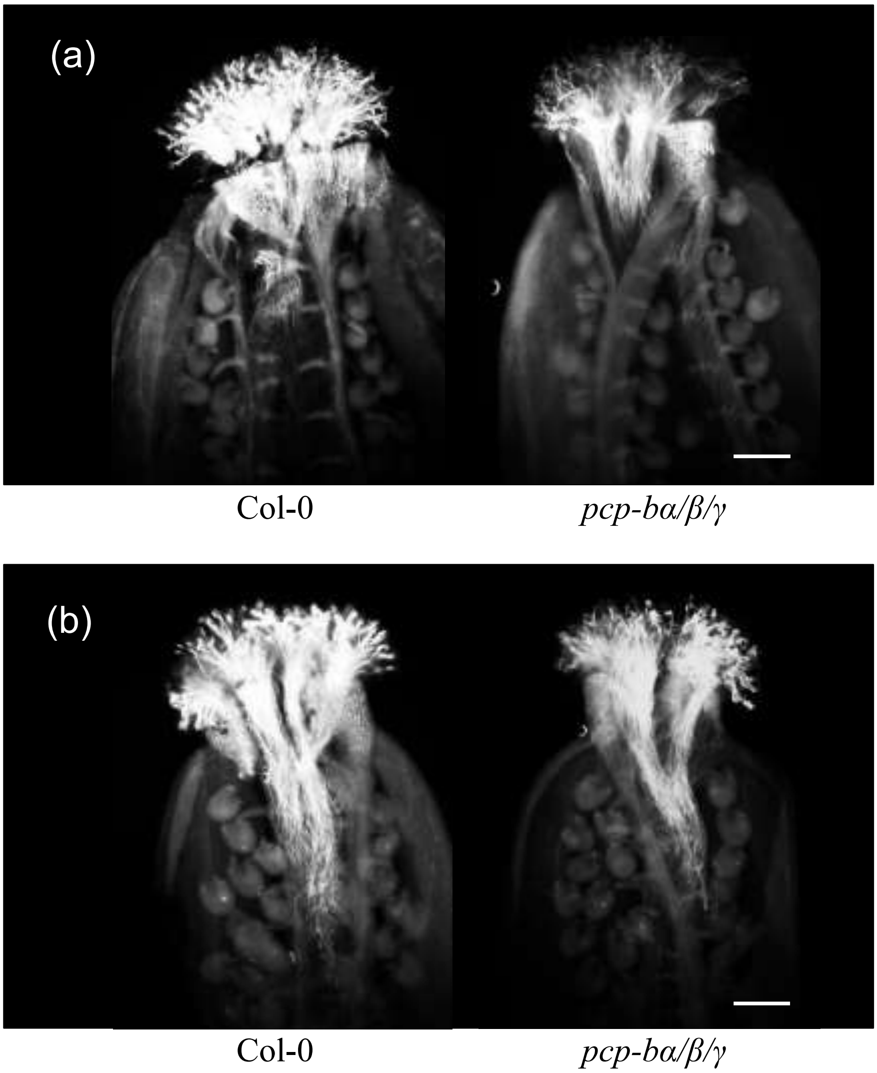

Fig. S10 Pollen tube growth comparisons of wild-type and *pcp-b* triple mutant after (a) 2 h and (b) 4 h of pollination. Bars, 0.2 mm.

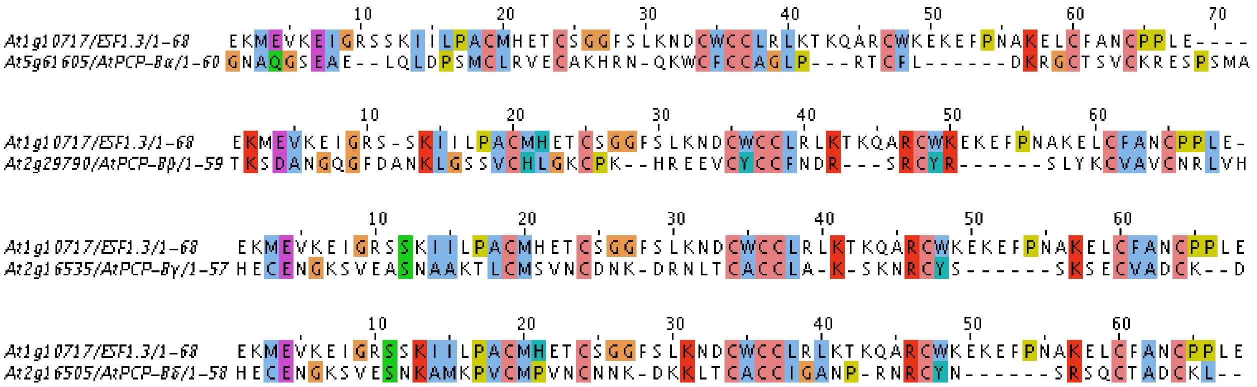

Fig. S11 Homologous alignments of ESF1.3 and AtPCP-Bs for protein structural predictions. Colour coding follows the default output for Clustal X (http://www.jalview.org/help/html/colourSchemes/clustal.html).

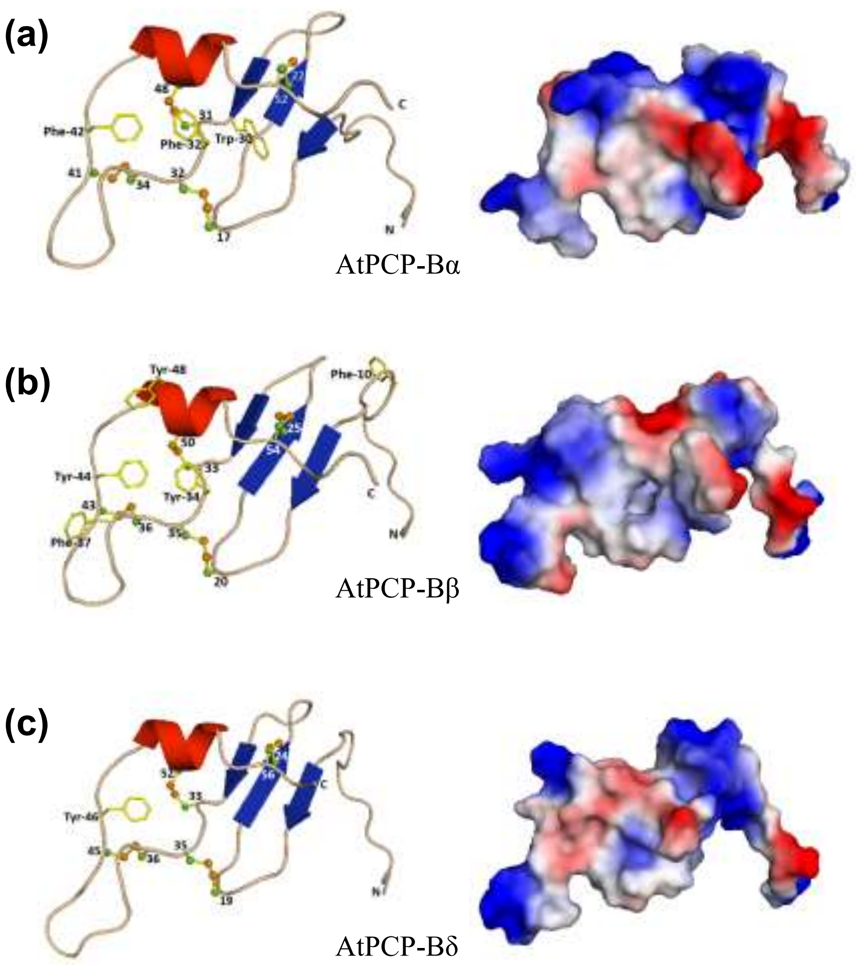

Fig. S12 Structural prediction models and surface distributions of electrostatic potential for AtPCP-Bs. (a) AtPCP-Bα, (b) AtPCP-Bβ and (c) AtPCP-Bδ predicted structures are displayed with both disulphide bonds and aromatic residues being indicated.

New Phytologist

Table S1 (a) PCR primers used for *PCP-B* homologous gene expression and T-DNA line genotyping, (b) *AtPCP-B* temporal gene expression pattern by RT-PCR and (c) genotyping of T-DNA mutant lines

(a)

Amplicon	Forward primer (5'-3')	Reverse primer (5'-3')
GapC (At3g04120)	TCAGACTCGAGAAAGCTGCTAC	GATCAAGTCGACCACACGG
At5g61605	TGCTATCCTTTGCCTCTTCATG	AGCCATTGAAGGAGATTCGCG
At2g29790	CAAGGTTTTGACGCGAACAAG	ATGAACAAGCCGGTTGCAG
At2g16535	CGCATCATCCTCATTTCTTC	ACACCTATTCTTGGATTTGGCC
At2g16505	GGGCCTTTGTATCATCCTG	TTGCAGTCAGCCGTACATTG
At2g41415	CCCATGCATATCTCGTCTGC	CAGCAATAACAGCCTCCGTC
At2g16225	TGTTCTCTCTCTTCGCTCTACA	CCGAAACAACACCAACAATG
At1g27135	TCCCTCTTCGCTATGCATGA	TCGAAGCAGCACCAACAATC
At5g50345	GCATCATCCTCGTTTCATTG	GGGTTAAGTCTAAGACACTC
At4g15953	TTCGTTTCCCTCTTTGGTGTGC	GGCCCTAATCCTTTGCTTACTC
At1g10747	ACTCATGCTCTCTCTCGTCG	CTTCCCAGCAAAGATCTGGC
At1g10745	CACAAACAGTTCTCATCTCC	AAACAACACCAACAATCCCG
At1g10717	ATCATGCTCTCCCTCTTTGCTC	GGCGAAACAAAGCTCCTTAG

(b)

Amplicon	Forward primer (5'-3')	Reverse primer (5'-3')
AtPCP-Bα (At5g61605)	TGCCTCTTCATGATTTTCCTCGTTCC	TTTATCAAGAAAACAGGTCCTCGG
AtPCP-Bβ (At2g29790)	GTAGTTTCTCTCGTTCCTCATGG	CGGTTGCAGACAGCCACACAC
AtPCP-Bγ (At2g16535)	CATCATCCTCATTTCTTCATTTCC	TTGCAGTCAGCAACACATTCTG
AtPCP-Bδ (At2g16505)	CATCCTGATTTCTTTCTTTCCTCTTC	GCCGTACATTGTGATCTGCTATTG
GapC (At3g04120)	CACTTGAAGGGTGGTGCCAAG	CCTGTTGTCGCCAACGAAGTC

(c)

Amplicon	Forward primer (5'-3')	Reverse primer (5'-3')
SALK_207087	GTTATGCCAATTCCAAAAGGC	TGCCTCTTCATGATTTTCCTC
SALK_062825	TTGAAATCCGAACCTGATTTG	TCTTATGGGGTTTTTGTGCAG
SALK_072366	TCCGTGGACTTGTGGTATACC	TTTCTTAATTCCTTAGTGGAGCTTG
GABI_718B04	TGGGACAGATTAAGAAGTTACGG	TGAAAACTCGTAGACCGCAAC
SALK line insertion left border	ATTTTGCCGATTTCGGAAC	-
GABI line insertion left border	ATAATAACGCTGCGGACATCTACATTTT	-

Table S2 Numbers and abbreviations of predicted PCP-B-like proteins in species and families

Family	Species	Protein names
Brassicaceae 160	*Arabidopsis thaliana* 12	AthB1-12
	Arabidopsis lyrata 14	AlyB1-14
	Capsella rubella 10	CarubB1-10
	Capsella grandiflora 12	CagraB1-12
	Capsella orientalis 13	CapOriB1-13
	Neslia paniculata 3	NespaB1-3
	Camelina sativa 4	CamSaB1-4
	Leavenworthia alabamica 4	LalaB1-4
	Boechera stricta 15	BostrB1-15
	Arabis alpina 3	AalB1-3
	Brassica oleracea 12	BoB1-11, BoPCP-B1
	Brassica rapa 9	BrapaB1-9
	Brassica napus 16	BnapB1-16
	Raphanus raphanistrum 6	RaphraB1-6
	Raphanus sativus 9	RaphsaB1-9
	Sisymbrium irio 3	SisirioB1-3
	Eutrema salsugineum 7	ThhalvB1-7
	Tarenaya hassleriana 8	TahassB1-8
Malvaceae 6	*Gossypium raimondii* 5	GoraiB1-5
	Gossypium arboreum 1	GoarbB1
Poaceae 91	*Oryza sativa* 2	OsB1-2
	Hordeum vulgare 26	HorvuB1-26
	Triticum urartu 4	TriurB1-4
	Aegilops tauschii 4	AtauB1-4
	Brachypodium distachyon 2	BdistaB1-2
	Zea mays 12	ZmB1-12
	Sorghum bicolor 14	SobicB1-14
	Panicum virgatum 3	PavirB1-3
	Panicum hallii 3	PahalB1-3
	Setaria italica 8	SetitaB1-8
	Oropetium thomaeum 2	OthoB1-2
	Eragrostis tef 11	EtefB1-11
Nelumbonaceae 14	*Nelumbo nucifera* 14	NenuB1-14
Pedaliaceae 1	*Sesamum indicum* 1	SeindB1
Solanaceae 8	*Nicotiana benthamiana* 8	NibenB1-8
Phrymaceae 2	*Mimulus guttatus* 2	MigutB1-2

New Phytologist

Table S3 Mean seed count values for Arabidopsis wild-type and *pcp-b* mutants

Genotype	Mean number of seeds per silique ± SD (n = 5)	P-value (Welsh's t-test)
Wile-type	60 ± 3	-
pcp-bα	62 ± 4	0.35
pcp-bβ	64 ± 5	0.92
pcp-bγ	55 ± 4	0.88
pcp-bδ	56 ± 4	0.92
pcp-bβ/γ	61 ± 5	0.98
pcp-bα/β/γ	62 ± 3	0.96

New Phytologist

Table S4 The global model quality estimation (GMQE) and qualitative model energy analysis (QMEAN4) scores of the predicted AtPCP-B protein models

Protein model	Sequence similarity	GMQE	QMEAN4
AtPCP-Bα	0.32	0.59	-5.52
AtPCP-Bβ	0.34	0.65	-4.74
AtPCP-Bγ	0.36	0.69	-6.14
AtPCP-Bδ	0.36	0.69	-5.37

Methods S1 Histochemical staining for β-glucuronidase (GUS) activity.

Leaves, open flowers and stage 12 buds of *pAt5g61605: GUS* and *pAt2g16505: GUS* lines were transferred into GUS substrate solution (100 mM sodium phosphate buffer pH 7.0, 10 mM EDTA, 0.1% v/v Triton X-100, 1mM potassium ferricyanide, 2 mM 5-bromo-4-chloro-3-indolyl-β-D-glucuronide) and vacuum-infiltrated for 5 min before overnight incubation at 37°C. Samples were then transferred into 50% (v/v) ethanol to remove chlorophyll and mounted in 50% (v/v) glycerol for imaging.
